# Comprehensive evaluation of complex polymicrobial specimens using next generation sequencing and standard microbiological culture

**DOI:** 10.1038/s41598-020-62424-x

**Published:** 2020-03-25

**Authors:** Lisa A. Cummings, Daniel R. Hoogestraat, Sara L. Rassoulian-Barrett, Christopher A. Rosenthal, Stephen J. Salipante, Brad T. Cookson, Noah G. Hoffman

**Affiliations:** 1 Departments of Laboratory Medicine, University of Washington, Seattle, Washington USA; 2 Departments of Microbiology, University of Washington, Seattle, Washington USA

**Keywords:** DNA sequencing, Next-generation sequencing, Diagnostic markers, Bacterial infection

## Abstract

Optimal clinical decision-making depends on identification of clinically relevant organisms present in a sample. Standard microbiological culture may fail to identify unusual or fastidious organisms and can misrepresent relative abundance of sample constituents. Culture-independent methods have improved our ability to deconvolute polymicrobial patient samples. We used next-generation 16S rRNA gene sequencing (NGS16S) to determine how often cultivatable organisms in complex polymicrobial samples are not reported by standard culture. Twenty consecutive bronchoalveolar lavage (BAL) samples were plated to standard and additional media; bacteria were identified by NGS16S analysis of DNA extracted directly from samples or from washed culture plates. 96% of organisms identified were cultivable, but only 21% were reported by standard culture, indicating that standard work-up provides an incomplete assessment of microbial constituents. Direct NGS16S correlated well with standard culture, identifying the same predominant organism in 50% of samples. When predominant organisms differed, NGS16S most often detected anaerobes, whose growth is unsupported by standard culture conditions for this specimen. NGS16S **i**dentified more organisms per sample and allowed identification of fastidious organisms, while culture was better at capturing organisms when bacterial load was low, and allowed incidental recovery of non-bacterial pathogens. Molecular and culture-based methods together detect more organisms than either method alone.

## Introduction

Culture is a “complex and difficult art”^1^. As the mainstay of the modern clinical microbiology laboratory, isolated growth of individual organisms is required for antimicrobial susceptibility and virulence testing, epidemiological investigations, and genome sequencing. Nevertheless, standard culture often fails to identify a causative pathogen when unusual or fastidious organisms are present, or after antimicrobial therapy has been initiated^2–9^. The developing field of culturomics has enabled the isolation of hundreds of new microorganisms, previously considered uncultivable, using a variety of growth conditions and extended incubation times^3,6,10–12^. For example, the addition of the antioxidant uric acid enables the aerobic growth of many organisms thought to be strictly anaerobic^13^. Therefore, we hypothesized that simple changes to routine culture conditions (for example, including additional types of growth media) could expand the repertoire of recoverable organisms in the clinical laboratory.

Next generation 16S rRNA gene sequencing (NGS16S) can be utilized for deconvolution of polymicrobial clinical samples that are difficult or impossible to resolve by standard molecular methods. Using synthetic polymicrobial samples of defined composition, we have shown that NGS16S analysis more accurately catalogs the bacterial contents of polymicrobial samples than standard culture^14^. However, this technology is expensive and requires technical expertise, limiting its routine use in the clinical laboratory. Here, we used NGS16S analysis of BAL samples, a readily accessible polymicrobial sample type, to evaluate the ability of culture to accurately catalog the microbial constituents of complex clinical samples. First, we analyzed DNA extracted directly from patient samples to determine the identity and prevalence of organisms for which current culture conditions are sub-optimal. In addition, we expanded standard culture by including four additional culture conditions, and evaluated washes of culture media plates by NGS16S analysis to determine the frequency with which cultivable organisms are not reported after standard work-up. We found that NGS16S identifies more organisms per sample and allows identification of fastidious organisms, while culture is better at capturing organisms when bacterial load is low and allows incidental recovery of non-bacterial pathogens such as yeast or molds. Both methods together detected more organisms in clinical samples than either method alone.

## Results

**Figure 1:**
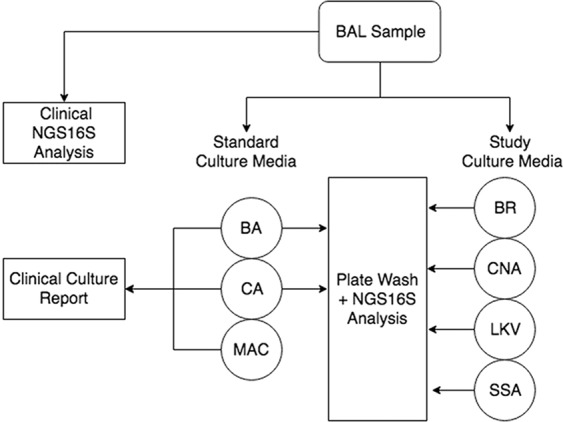
Study Schematic. The constituents of each BAL sample were surveyed by standard microbiological culture (BA, CA, MAC, incubated at 37 °C for 3 days) and NGS16S sequencing of DNA extracted directly from the sample. Samples were also plated to study media (BR, CNA, LKV and SSA) and incubated anaerobically for 7 days. Individual PBS plate washes were analyzed by NGS16S sequencing.

Twenty consecutive BAL samples were collected from in-house oncology or transplant patients. Two samples (BAL03 and BAL06) were from the same patient; all other samples were collected from different individuals. Specimens were analyzed as outlined in Fig. 1. Standard clinical microbiological culture and NGS16S sequencing of DNA extracted directly from clinical specimens (direct NGS16S) was performed by the UWMC clinical microbiology laboratory as described in the methods. No bacteria were reported by standard microbiological work-up for six samples (BAL 06, 07, 11, 12, 15 and 18; Table 1). No reportable organisms were detected by direct NGS16S analysis for four samples (BAL 07, 11, 15 and 18; Table 2, Supplementary Table S1**)**. An equal volume of each specimen was also plated to study culture media (BR, CNA, LKV, and SSA, see methods) and incubated anaerobically for seven days. Standard and study culture plates were washed with PBS and organisms present were identified by NGS16S analysis (Fig. 1).

**Table 1:** Clinical culture results.

Specimen	Reported result	Classification group (CRC)	Quantification^a^
BAL01	viridans streptococci	viridans streptococci	1+
BAL02	*Staphylococcus aureus*	*Staphylococcus aureus*	2+
	*Rothia mucilaginosa*	*Rothia* species	2+
	viridans streptococci	viridans streptococci	2+
	*Actinomyces* species	*Actinomyces* species	1+
	*Dermacoccus* species	*Dermacoccus* species	1 colony
BAL03	viridans streptococci	viridans streptococci	>10000 col/ml
	*Haemophilus parainfluenzae*	*Haemophilus* species	400 col/ml
BAL04	*Actinomyces odontolyticus*	*Actinomyces* species	2100 col/ml
	*Rothia* species	*Rothia* species	700 col/ml
	viridans streptococci	viridans streptococci	100 col/ml
BAL05	*Staphylococcus*, coagulase negative	*Staphylococcus*, coagulase negative	300 col/ml
BAL06	NEGATIVE		
BAL07	NEGATIVE		
BAL08	*Actinomyces odontolyticus*	*Actinomyces* species	4+
	*Rothia* species	*Rothia* species	4+
	*Streptococcus mitis* group	viridans streptococci	4+
	*Streptococcus parasanguinis*	viridans streptococci	4+
BAL09	viridans streptococci	viridans streptococci	10500 col/ml
	*Haemophilus parainfluenzae*	*Haemophilus* species	7500 col/ml
	*Neisseria* species	*Neisseria* species	6000 col/ml
	*Rothia mucilaginosa*	*Rothia* species	2000 col/ml
BAL10	*Aspergillus fumigatus*	Yeast/fungi	10 colonies
	Contaminating oral microbiota	viridans streptococci	3+
BAL11	NEGATIVE		
BAL12	NEGATIVE		
BAL13	*Actinomyces odontolyticus*	*Actinomyces* species	1+
	viridans streptococci	viridans streptococci	1+
BAL14	*Staphylococcus aureus*	*Staphylococcus aureus*	>10000 col/ml
	nonhemolytic streptococci	viridans streptococci	5000 col/ml
	*Rothia* species	*Rothia* species	5000 col/ml
	viridans streptococci	viridans streptococci	5000 col/ml
BAL15	NEGATIVE		
BAL16	viridans streptococci	viridans streptococci	5300 col/ml
	*Neisseria* species	*Neisseria* species	5100 col/ml
	aerobic non-sporeforming Gram-positive rods	aerobic non-sporeforming Gram- positive rods	700 col/mL
	*Bacillus* species, not *Bacillus anthracis*	*Bacillus* species	100 col/ml
BAL17	nonhemolytic streptococci	viridans streptococci	200 col/ml
	viridans streptococci	viridans streptococci	200 col/ml
BAL18	NEGATIVE		
BAL19	viridans streptococci	viridans streptococci	4+
	*Neisseria* species	*Neisseria* species	3+
	*Rothia mucilaginosa*	*Rothia* species	3+
	*Haemophilus parainfluenzae*	*Haemophilus* species	2+
BAL20	viridans streptococci	viridans streptococci	500 col/ml
	*Rothia* species	*Rothia* species	200 col/ml
	*Haemophilus parainfluenzae*	*Haemophilus* species	100 col/ml
	*Neisseria* species	*Neisseria* species	20 col/ml

^a^routine culture requests were quantified on a 1+ to4+ scale; if quantitative culture was ordered, quant is given as col/mL.

**Table 2:** Specimen Classification Details^a^.

Specimen	Total	Reported	NGS16S	Standard media^b^	Study media^c^	BR	Load^d^	Complexity^e^
BAL01	4	1	2	1	4	3		
BAL02	23	5	6	10	17	17		High
BAL03	8	2	3	5	7	6		
BAL04	7	3	4	5	4	4		
BAL05	5	1	2	2	4	3		
BAL06	1	0	1	0	0	0	Low	
BAL07	1	0	0	0	1	0	Low	
BAL08	21	4	5	3	20	10		High
BAL09	20	4	8	7	17	13		High
BAL10	10	2	6	2	9	8		High
BAL11	2	0	0	0	2	0	Low	
BAL12	4	0	3	0	1	0	Low	
BAL13	11	2	8	2	11	10		High
BAL14	10	4	3	4	9	6		High
BAL15	4	0	0	1	2	2	Low	
BAL16	17	4	8	7	12	12		High
BAL17	13	2	6	2	9	5		High
BAL18	0	0	0	0	0	0	Low	
BAL19	22	4	10	8	20	17		High
BAL20	15	4	7	5	13	11		High

^a^Number of unique CRCs in each category is specified.

^b^BA and CA.

^c^BR, CNA, LKV, and SSA.

^d^Low bacterial load = no bacteria reported from standard culture.

^e^High complexity = 10 or more CRCs identified in a specimen.

Thirty-nine clinically relevant classifications (CRCs) were identified in this study either by identification of colonies in standard clinical culture workup or by NGS16S sequencing as described in the methods (Table 3). CRCs commonly reported by standard culture of BAL samples from the general patient population were compared to our study CRCs. Six of the 10 most abundant CRCs in our study were anaerobes whose growth is unsupported by standard culture conditions. Common CRCs observed among historical specimens not recovered in our study included enteric Gram-negative rods, *Enterococcus* species and *Pseudomonas* species. This is likely due to the small sample size of our study, and sampling restricted to transplant and oncology patients.

**Table 3:** Organisms detected in this study.

Classification group (CRC)^a^	Study prevalence (%)^b^	Historic prevalence (%)^c^
*Actinomyce*s species*	15 (75)	27 (6.6)
*Prevotella* species*	14 (70)	
viridans streptococci	14 (70)	126 (30.7)
*Veillonella* species*	13 (65)	
aerobic non-sporeforming Grampositive rods	12 (60)	32 (7.8)
*Rothia* species	11 (55)	66 (16.1)
*Campylobacter* species*	10 (50)	
*Atopobium* species*	7 (35)	
*Capnocytophaga* species*	7 (35)	3 (0.7)
*Staphylococcus*, coagulase negative	7 (35)	96 (23.4)
*Haemophilus* species	6 (30)	31 (7.5)
*Lachnoanaerobaculum* species*	6 (30)	
*Lactobacillus* species*	6 (30)	47 (11.4)
*Megasphaera* species*	6 (30)	
*Solobacterium* moorei*	6 (30)	
*Fusobacterium* species*	5 (25)	
*Oribacterium* species*	5 (25)	
*Staphylococcus aureus*	5 (25)	38 (9.2)
*Neisseria* species	4 (20)	36 (8.8)
*Selenomonas* species*	4 (20)	
*Dermacoccus nishinomiyaensis*	3 (15)	
*Porphyromonas* species*	3 (15)	
*Stomatobaculum* species*	3 (15)	
*Alloprevotella* species*	2 (10)	
*Dialister* species*	2 (10)	
*Leptotrichia* species*	2 (10)	
Parvimonas micra*	2 (10)	
*Peptostreptococcus* species*	2 (10)	
[Eubacterium] sulci*	1 (5)	
[Eubacterium] yurii*	1 (5)	
Anaeroglobus species*	1 (5)	
Bacillus species, not *Bacillus anthracis*	1 (5)	1 (0.2)
*Dermabacter* species	1 (5)	
*Dermacoccus* species	1 (5)	
*Eikenella corrodens*	1 (5)	2 (0.5)
Marmoricola aurantiacus*	1 (5)	
*Peptoanaerobacter* species*	1 (5)	
Skermanella aerolata*	1 (5)	
*Tropheryma whipplei*	1 (5)	
Yeast/fungi	1 (5)	49 (11.9)
Enteric Gram-negative rod		48 (11.7)
*Enterococcus* species		20 (4.9)
*Pseudomonas* species		17 (4.1)

^a^To facilitate comparisons between culture and NGS16S analysis, organisms were assigned to classification groups as detailed in Table 1 and Supplementary Table S1. Anaerobes are noted with an*.

^b^Number of positive study specimens (total number of specimens = 20); positive is defined as reportable by standard culture or NGS16S and/or identified in at least one culture plate wash.

^c^Number of positive historical specimens (total number of specimens = 411); positive = reported in standard culture.

Bacteria were defined as present in any given specimen if any one of the following criteria was met: (1) reported in standard culture; (2) read mass detected in one or more culture plate washes above filtering threshold (see methods); (3) reportable by direct NGS16S analysis. If either of the former two criteria were met, the organism was considered cultivable. Eleven of the 39 CRCs identified in this study (28%) were reported from standard culture at least once and 35 (90%) were recovered on at least one culture plate (16 from standard media and 31 on study media, Table 4). Thus, the majority of organisms identified in this study were cultivable. Twenty of the 28 CRCs identified in more than one specimen were anaerobes (Table 4), and anaerobes were the predominant classification by direct NGS16S for 7/20 samples (35%, Table 5).

**Table 4:** Detection method details.

Category	Classification group (CRC)^a^	Number of samples					
		Prevalence^b^	Reported by standard culture	Reportable by direct NGS16S^c^	Plate wash positive by NGS16S		
					Any	Study^d^	Standard^e^
Current culture conditions sufficient for detection	viridans streptococci	14	13	13	13	12	13
	*Staphylococcus aureus*	2	2	2	2	2	2
	*Neisseria* species	4	4	4	4	1	4
	*Bacillus* species, not *Bacillus anthracis*	1	1		1		1
Current culture conditions insufficient for detection	*Prevotella* species*	14		10	14	14	
	*Veillonella* species*	13		12	13	13	3
	aerobic non-sporeforming Gram-positive rods	11	1		12	10	2
	*Campylobacter* species*	10		6	10	10	
	*Atopobium* species*	7			7	7	
	*Capnocytophaga* species*	7		2	6	5	1
	*Lachnoanaerobaculum* species*	6			6	6	
	*Lactobacillus* species*	5			5	5	
	*Megasphaera* species*	6		3	5	5	
	*Solobacterium* moorei*	6		1	6	6	
	*Fusobacterium* species*	5		3	4	4	1
	*Oribacterium* species*	5			5	5	
	*Selenomonas* species*	4			4	4	
	*Porphyromonas* species*	3		3	3	3	1
	*Stomatobaculum* species*	3			3	3	
	*Alloprevotella* species*	2		1	2	2	
	*Dialiste*r species*	2			2	2	
	*Leptotrichia* species*	2		1	2	2	
	Parvimonas micra*	2			2	2	
	*Peptostreptococcu*s species*	2			2	2	
	[Eubacterium] sulci*	1			1	1	
	[Eubacterium] yurii*	1			1	1	
	*Anaeroglobus* species*	1			1	1	
	Marmoricola aurantiacus*	1		1			
	*Peptoanaerobacter* species*	1			1	1	
	Skermanella aerolata*	1		1			
Poor correlation between culture report and growth on standard culture media	*Actinomyces* species*	15	4	8	15	15	8
	*Rothia* species	11	7	2	11		11
	*Staphylococcus*, coagulase negative	7	1	0	7	5	4
	*Haemophilus* species	6	4	5	6	3	6
	*Dermacoccus* species	3	1		3		3
	*Dermabacter* species	1			1		1
	*Eikenella corrodens*	1			1	1	1
Special case organisms	Yeast/fungi	1	1				
	*Tropheryma whipplei*	1		1			

^a^To facilitate comparisons between culture and NGS16S analysis, organisms were assigned to classification groups as detailed in Table 1 and Supplementary Table S1. Anaerobes are noted with an*.

^b^Number of positive study specimens (total number of specimens = 20); positive is defined as reportable by standard culture or NGS16S, and/or recovered in at least one culture plate wash.

^c^as evaluated by UWMC Clinical Microbiology; note that in some cases reads may be detected but fall below reporting thresholds.

^d^BR, CNA, LKV, SSA.

^e^BA or CA.

**Table 5:** Correlation of Gram stain, standard culture and direct NGS16S.

Specimen	Gram Stain^a^		Predominant by culture^b^	Predominant by direct NGS16S^c^
	PMN	Organisms		
BAL01	1+		viridans streptococci	viridans streptococci
BAL02	2+	Rare GPC	viridans streptococci	*Veillonella* species
BAL03	4+	3+ GPC	viridans streptococci	viridans streptococci
BAL04	1+		*Actinomyces odontolyticus*	*Prevotella* species*
BAL05			*Staphylococcus*, coagulase negative	*Prevotella* species*
BAL06	3+		no growth reported	viridans streptococci
BAL07	1+		no growth reported	no organisms detected
BAL08	2+	1+ GPC	viridans streptococci	*Veillonella* species/*Prevotella* species*
BAL09	2+	2+GPC/2+GNR	*Haemophilus parainfluenza*	*Haemophilus* species
BAL10	2+	1+ GPC	*Aspergillus*	viridans streptococci
BAL11			no growth reported	no organisms detected
BAL12	3+		no growth reported	*Megasphaera* species*
BAL13			viridans streptococci	*Prevotella* species*
BAL14	2+	2+ GPC	*Staphylococcus aureus*	*Staphylococcus aureus*
BAL15	2+		no growth reported	no organisms detected
BAL16	rare		viridans streptococci	viridans streptococci
BAL17	3		viridans streptococci	*Tropheryma whipplei*
BAL18	2+	Rare GPC	no growth reported	no organisms detected
BAL19	rare	1+ GPC/1+ GNR	*Neisseria/Rothia*	*Prevotella* species*
BAL20	1+		viridans streptococci	viridans streptococci

^a^PMN = Polymorphonuclear neutrophils; GPC = Gram positive cocci, GNR = Gram negative rods.

^b^See Table 1 for all culture results.

^c^See Supplementary Table S1 for all NGS16S results. Anaerobes are noted with an*.

**Figure 2:**
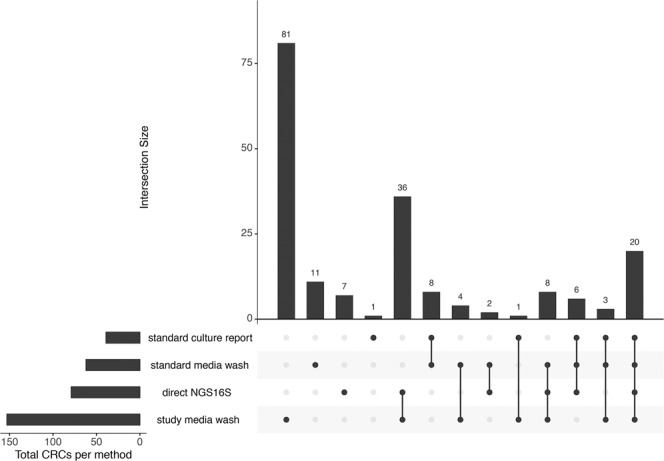
Classification assignment details. The number of CRC assignments attributed to each combination of survey methods (Fig. 1) is shown; dots below each bar indicate methods resulting in detection. Standard media =BA and/or CA; direct NGS16S = NGS16S analysis of DNA extracted directly from clinical samples; study media = BR, CNA, LKV and/or SSA. Figure was generated using UpSetR (10.1093/bioinformatics/btx364).

A total of 188 CRC assignments were made among the 20 specimens, of which 181 (96%) were cultivable (Fig. 2, Table 4). Only 39 (21%) assignments were reported from standard culture and 79 (42%) were reportable by direct NGS16S analysis. Thus, neither method alone was able to provide a complete assessment of the organisms present in the patient samples. Sixty-two (33%) of the assignments were detected in standard culture plate washes and 157 (90%) were detected in study culture plate washes (Fig. 2, Table 4). Together, these data suggest many organisms present in these specimens either (1) have culture requirements not currently met by standard culture conditions and/or (2) were viable on current culture media but failed to be identified by standard culture work-up.

The CRC assignments fell into four categories based on comparisons of culture report, direct NGS16S analysis, and NGS16S plate wash analysis (Table 4). Two organisms were special cases: *Aspergillus*, recovered from BAL10, is not detectable by NGS16S analysis (which is restricted to bacterial 16S rRNA), and *Tropheryma whipplei*, detected by direct NGS analysis in BAL17, requires very specialized growth conditions. Twenty-one assignments of viridans streptococci, *Staphylococcus aureus*, *Neisseria* species and *Bacillus* species constituted a group for which current culture conditions were sufficient for detection and reporting.

The largest category comprised a total of 121 assignments of 26 CRCs for which current culture conditions were insufficient for detection (i.e., not reported in standard culture and/or not detected in BA or CA plate wash by NGS16S analysis). This category represents a majority (26/39) of all CRCs and 6 of the 10 most prevalent classification groups (Tables 4 and 5). Forty-four assignments in this group were reportable by NGS16S. However, the remaining assignments were of low relative abundance: 66 assignments were detected in samples below the 1% relative abundance reporting threshold, and the remaining 11 assignments were not detected by direct NGS16S in any amount (data not shown). Culture was the more reliable detection method for these low abundance assignments (Table 4).

A final category includes a total of 44 assignments of seven CRCs for which there was poor correlation between the CRCs report from standard culture and growth on standard media as measured by NGS16S plate wash analysis (Tables 4 and 6). This discrepancy indicates that organisms capable of growth under standard culture conditions may fail to be identified during culture work-up. Most of these CRCs were in low abundance (0–4.7% relative abundance by direct NGS16S analysis) from specimens containing 10 or more CRCs (Tables 4 and 6). One specimen (BAL10) was overgrown with *Aspergillus*, making isolation of bacterial colonies difficult. In two specimens (BAL02 and BAL14) the predominant organism was *Staphylococcus aureus;* the presence of a known pathogen with a distinct colony morphology may have resulted in a less rigorous examination of the plates for additional colony types. *Rothia* species were frequently reported by standard culture and identified from plate washes of standard culture media. However, this classification was infrequently considered reportable by direct NGS16S **(**Table 4**)**. An alignment of the universal NGS16S primer sequence against the 16S sequences of *Rothia mucilaginosa* type strains revealed a single nucleotide mismatch, which could affect relative amplification and account for the lower than expected reporting rate of this species from direct specimens.

**Table 6:** CRCs detected on standard culture media but not reported.

Specimen	Classification group (CRC)^a^	NGS16S % abundance (Direct)	Detected on plate wash					
			BA^b^	CA^b^	BR^c^	CNA^c^	LKV^c^	SSA^c^
BAL03	*Actinomyces* species	0.24		X	X	X		X
BAL16	*Actinomyces* species	1.32		X	X	X		X
BAL19	*Actinomyces* species	0.74	X	X	X	X		X
BAL20	*Actinomyces* species	2	X	X	X	X		X
BAL17	*Dermabacter* species	0		X				
BAL15	*Dermacoccus* species	0		X				
BAL16	*Dermacoccus* species	0		X				
BAL19	*Eikenella corrodens*	0.09		X	X			
BAL02^d^	*Haemophilus* species	0.62	X	X				
BAL16	*Haemophilus* species	2.62		X				
BAL03	*Rothia species*	0.86	X	X				
BAL05	*Rothia species*	0		X				
BAL10^e^	*Rothia species*	4.66		X				
BAL16	*Rothia species*	0.68	X	X				
BAL02	*Staphylococcus*, coagulase negative	0.73		X				
BAL09	*Staphylococcus*, coagulase negative	0.44	X	X				
BAL14^d^	*Staphylococcus*, coagulase negative	0	X			X		

^a^To facilitate comparisons between culture and NGS16S analysis, organisms were assigned to classification groups as detailed in Table 1 and Supplementary Table S1.

^b^Standard media.

^c^Study media.

^d^Predominant *Staphylococcus aureus* reported.

^e^Overgrown with *Aspergillus*.

Standard culture returned an average of 2.1 CRCs/specimen (range 0–5, Table 2), while plate washes of standard culture media returned an average of 3.2 CRCs (range 0–10, Table 2). The fact that the unreported CRCs present on standard culture media were most frequently identified in samples of high complexity (10 or more organisms, Table 2) indicates the challenges of accurately discerning colony type subpopulations in complex polymicrobial samples on plated media, and suggests that recovery of rare organisms is reduced when more than 5 types are present. Direct NGS16S returned an average of 9.6 CRCs/specimen (range 0 to 22, Table 2) demonstrating the superior ability of this method for deconvoluting polymicrobial samples. Standard culture and direct NGS16S identified the same predominant CRC (or lack of organisms) for 10/20 samples (Table 5).

**Figure 3:**
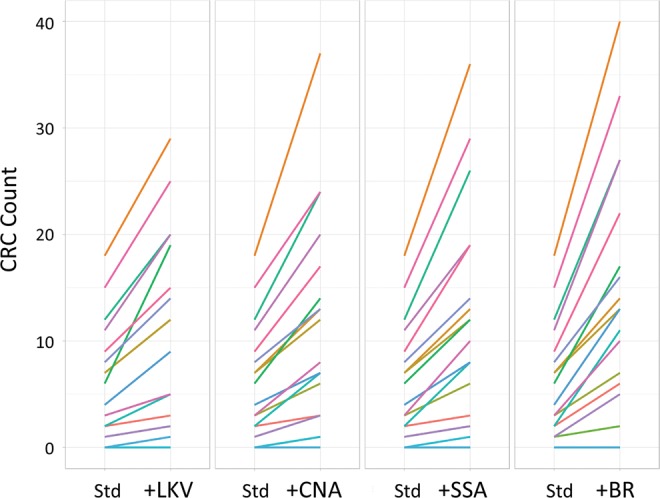
Value-added media assessment. The effect of adding a single additional study medium to current standard media for each specimen is shown. The cumulative count of CRC assignments increases as new CRCs are identified. Std = current culture media (BA + CA).

**Figure 4:**
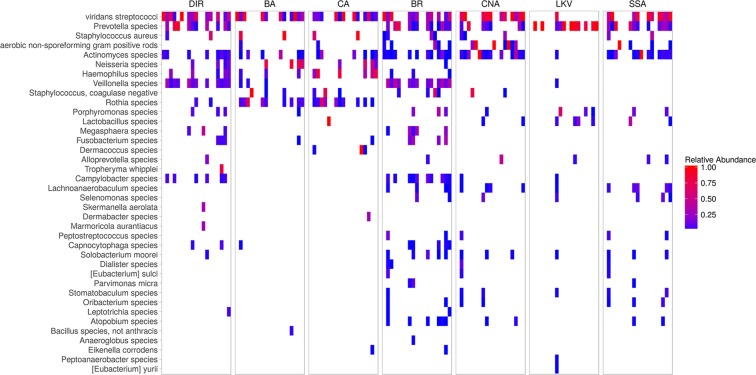
CRC heat map. Relative abundance of each classification group for each of the 20 specimens is shown by media type; relative abundance is indicated by color. DIR = NGS16S sequencing of DNA extracted directly from the sample; plate washes from media as described in Fig. 1.

Given that we estimate 96% of reportable organisms present in samples are cultivable but only 21% are reported, standard conditions are clearly sub-optimal and could potentially be improved. Because the majority of organisms were detectable on one or more study plates, we evaluated the value of supplementing current culture conditions with a single additional medium. First, we compared the number of CRCs detected by plate wash for standard culture media with or without each study medium individually (Fig. 3). Next, we compared the relative abundance per sample of all CRCs on each media type to identify the CRCs supported by each (Fig. 4). All study media provided an increase in the number of organisms detected per specimen; BR provided the largest advantage for the greatest number of specimens (Fig. 3) and supported the greatest number of different organisms (Fig. 4). As predicted, selective media that suppressed the growth of many organisms increased the relative abundance of target organisms (for example LKV and SSA preferentially supported *Prevotella* species and viridans streptococci, respectively (Fig. 4, Supplementary Fig S1).

## Discussion

A comprehensive description of the microbial constituents of complex samples has an immediate application in the clinical management of patients when organisms with known pathological properties are identified, or when such organisms can be ruled out. In addition, a more complete profile of the bacterial population present in a specimen may provide additional information as medical knowledge advances: individual organisms or constellations of organisms may serve as biomarkers of disease^15–19^, or distinct microbial profiles may be associated with different disease states or prognoses^20–22^. While NGS and other emerging technologies can directly expand the resolution of bacterial detection and classification in clinical specimens, there are also opportunities to improve existing methodologies. This study explored possible improvements to routine clinical practices for complex polymicrobial specimens using NGS16S analysis as a benchmark. We chose BAL specimens for our study because they are both readily accessible and likely to be polymicrobial. We restricted our study to samples collected from oncology/transplant patients who are particularly susceptible to lower respiratory infections which are frequently polymicrobial and/or involve organisms not traditionally thought of as pathogens^7,23,24^.

In general, there was good correlation between NGS16S and standard culture results, with the same predominant organisms identified by both methods in half of the samples (Table 5). In 7 of the cases where different predominant organisms were identified, NGS16S identified anaerobes (Table 5), consistent with the fact that anaerobes are not routinely cultured for this specimen type. *Prevotella* and *Veillonella* were frequently recovered (14/20 samples, Table 3) and represented the most abundant CRC in 6/20 samples (Table 5), consistent with previous studies^14,25–27^. Anaerobes were the predominant organism in approximately one third of the samples overall (Table 5). Anaerobes may contribute to pathogenicity in the lung either directly or indirectly via the production of beta-lactamases or other secreted factors or by interactions with other members of the lung microbiota^26,28–35^. Anaerobes have also been recovered in high abundance in BAL from cystic fibrosis patients with high antibody titers, providing evidence that these organisms can be present in sufficient abundance and duration to trigger host immune responses^29,36,37^. Although not traditionally thought of as respiratory pathogens, these data suggest that anaerobes may be a significant constituent of the lung microbiome and further studies on their contribution to respiratory pathogenesis are warranted.

Although anaerobes were the organisms most frequently missed by standard culture, other cultivable organisms, many of which are members of the oral microbiota, were also overlooked **(**Tables 4 and 6). The clinical significance of this finding is undetermined, although underappreciated contributions to disease of “normal” microbiota, are documented in the literature^32,38^. The lungs are not, in fact, sterile^39^, and the lung microbiota most likely originates from micro-aspiration of the oral microbiota^39,40^. Thus, identification of such organisms is not unexpected. Although incidental contamination from oral microbiota during sample collection is possible, many recent studies indicate that this is unlikely to be a significant source of organisms identified from BAL specimens^39–44^. Clinical context is also an important factor when interpreting the significance of any given organism present in a sample. The list of canonical pathogens for the same specimen type may differ based on patient population, and expanding this list to include non-conventional organisms may improve patient care in some circumstances. Our study highlights the fact that cultivatable organisms, often present in major abundance, are frequently missed by standard culture and supports the idea that until a comprehensive catalog of complex samples is routinely attainable, the list of clinically relevant organisms for a sample type cannot be definitively defined. One can’t evaluate the importance of what one doesn’t know is there.

Although optimal culture conditions to recover all organisms present in a BAL is likely to be patient specific^11^, we identified BR as the best single value-added media, supporting the growth of the largest number of organisms for most specimens (Figs. 3–4). However, selective media was often better than BR for the recovery of certain organisms. For example, *Prevotella*, the second most frequently isolated CRC (Table 3), was predominant on LKV plates (Fig. 4, Supplementary Fig. S1). This illustrates a fact well known to microbiologists: no single culture medium meets all needs. By design, selective media support the growth of target organisms only; rich media supports the growth of many organisms that could outcompete slow growers or obscure the presence of small colony types, especially when in low abundance^14^. The physical isolation and differentiation of individual colonies on a culture plate becomes  increasingly difficult as the bacterial load or the number of colony types in the sample increases^6,14^. This is illustrated by failure to identify all cases of currently “growable” organisms like *Haemophilus* which was detected by NGS16S on standard culture plates (Table 4); this phenomenon has been observed in other studies^3,6,18^. In addition, culture plates with a predominance of normal microbiota or a known pathogen such as *Staphylococcus aureus*, may be less carefully scrutinized by laboratory personnel, increasing the likelihood that rare and/or small colony types are overlooked. Our study indicates that five colony types on a single culture plate is the functional limit. Together these data suggest that while the addition of BR to routine culture set-up will broaden the spectrum of recoverable organisms for this sample type to include anaerobes, isolation of any but the most predominant colony types may still be challenging.

Although similar information can theoretically be obtained by either culture or direct NGS16S^14^, molecular methods allow direct identification of organisms that may require prior knowledge of specialized culture conditions, increasing the ability to detect unusual organisms. This was the case for BAL17, where *Trophyrema whipplei*, a highly fastidious organism that can cause acute pneumonia^45,46^, was detected as the predominant organism. Additionally, direct NGS16S often has a lower turnaround time than culture^2^, particularly for slower growing organisms or those that need subculture for biochemical testing. Although bioinformatic support is required to analyze the results of molecular testing, generating and sequencing next-generation libraries is often straightforward and requires less training than culture.

Multiple factors influence the ability of various methods to detect organisms present in a clinical sample: the overall bacterial load, the number and relative abundance of individual organisms present, as well as organism-specific growth conditions, colonial morphology and DNA extraction efficiency. Molecular methods do an excellent job of de-convoluting highly polymicrobial samples, especially when bacterial load is high. Culture was more sensitive than NGS16S for capturing low-abundance organisms, particularly when bacterial load is too low for efficient PCR amplification (Table 4), and allows incidental recovery of non-bacterial pathogens such as yeast or molds. Combining molecular and culture-dependent methods increases the sensitivity of detection compared to either method alone^11,18,47^. Therefore, culture and 16S sequencing should be used together for the most comprehensive evaluation of complex polymicrobial specimens^27^.

## Methods

### Sample collection

Twenty consecutive BAL samples were prospectively collected from in-house oncology or transplant patients. Participants were identified based on hospital ordering location and specimen type, without any other selection or eligibility criteria. Use of clinical microbiological specimens was approved by the University of Washington Human Subjects Review Board (approval number 42541). Specimens were fully de-identified after being aliquoted from the material submitted for clinical testing, and as such this study does not constitute human subjects research according to University of Washington Institutional Review Board criteria. All experiments were performed in accordance with relevant guidelines and regulations. A 2 mL aliquot of each BAL sample was frozen immediately after culturing and stored at −80 °C until DNA extraction.

### Microbiological culture

Standard microbiological culture was performed by the University of Washington Clinical Microbiology Laboratory, as previously described^48^. Briefly, samples were plated on 5% sheep blood (BA), MacConkey (MAC) and chocolate (CA), agar plates (standard media), and incubated aerobically at 37 °C for 72 h. An internal review of all organisms reported from BAL by clinical NGS16S analysis in our institution identified organisms for which standard culture conditions may be inadequate; additional media was selected for ability to support the growth of these organisms (study media). Brucella agar (BR, Remel) is a general purpose anaerobic medium. Laked Sheep Blood with Kanamycin and Vancomycin Agar (LKV, Hardy Diagnostics) is used for the selective isolation of fastidious and slow growing Gram-negative obligately anaerobic bacteria. Selective Strep Agar (SSA, Hardy Diagnostics) is designed to inhibit Gram-negative bacilli and *Staphylococci*, thereby allowing the isolation and identification of pathogenic streptococci, including beta-hemolytic streptococci and *Streptococcus pneumoniae*. Columbia CNA Agar (CNA, Remel) was designed to suppress the growth of most Gram-negative bacteria, thus enriching for Gram-positive bacteria. In addition to standard media, 0.1 mL of each specimen was plated to study media and incubated anaerobically at 37 °C for seven days. Photographs were taken of all plates at the end of incubation.

### DNA extraction, library preparation, and sequencing

All culture plates except MAC (regardless of visible bacterial growth) were washed with 3 mL sterile PBS and bacterial colonies were released by gently scraping agar surface with a sterile cell scraper. Bacteria from 1 mL of the resulting suspension were collected by centrifugation, resuspended in 0.2 mL MagNA Pure DNA Tissue Lysis Buffer (Roche) and stored at −80 °C until extraction. DNA was extracted from patient samples and plate washes using the QIAamp UCP Pathogen Mini Kit (Qiagen) with mechanical disruption of samples with 1.4-mm ceramic beads followed by enzymatic lysis via proteinase K. Next generation sequencing libraries were prepared and DNA sequencing was performed as previously described^14^. Briefly, the 16S v1–v2 region was amplified using custom primers incorporating Illumina-compatible sequencing adaptors and a sample-specific 8-bp barcode sequence; paired-end sequencing was performed on an Illumina Miseq using a 500-cycle sequencing kit (version 2) to a minimum read depth of 50,000 reads per sample. Sequence data generated for this study have been submitted to the NCBI Sequence Read Archive (SRA) under accession no. PRJNA555084.

### Data analysis

NGS16S analysis of DNA extracted from patient samples (direct NGS16S) was performed by the University of Washington Clinical Microbiology Laboratory. Sequence analysis was performed without knowledge of culture results. Briefly, sample sequences were demultiplexed into paired end sequence fastq files using the Illumina on-board software with barcodes and adapters removed. Sequence variants (SVs) were generated from the paired end sequence fastq files using DADA2^49^. SVs were identified as 16S rRNA by multiple sequence alignment using cmsearch^50^ using the default settings and a covariance model available from the Infernal web site (http://infernal.janelia.org). SVs were then passed through the decontam software package^51^ to identify and remove contaminants. To reduce the effects of possible DNA carry over between runs or samples, SVs corresponding to 100 reads or fewer in each sample were excluded. The remaining SVs were used as blast queries against a curated set of 16S rRNA records retrieved from NCBI. Alignments of at least 90% query coverage were grouped taxonomically and classified as previously described^14^. An *Acinetobacter* species SV present in 100% of samples analyzed was used as an internal standard to calculate the number of templates for each classification. All reads were classified, and classifications >1% of the total specimen read mass were considered reportable. In three cases, biologically relevant organisms were only slightly below this threshold and were also included: *Actinomycyes odontolyticus* was included for BAL01 and BAL02 (0.72% and 0.89% raw reads, respectively) and *Solobacterium moorei* for BAL13 (0.95% raw reads). Plate wash reads were processed as described above, with additional filtering steps: (1) all sequences with fewer templates than the internal standard were removed from each sample as likely reagent background, and (2) the number of templates expected to produce a visible colony was empirically determined (2500 templates) and SVs below this threshold were excluded from further analysis. Five classification assignments that were not excluded by these filtering criteria were manually excluded from analysis as contaminating DNA; these were recovered from plates without corresponding colonies and were near the filtering threshold. On average, 98% of total reads from plates with bacterial growth were analyzed (range 83–99.9%); all reads from no-growth plates were excluded, confirming that filtering was appropriately removing irrelevant sequences. To compare standard clinical lab culture and NGS16S results, organisms were manually combined into clinically relevant classifications (CRCs) at genus level or based on similar taxonomy and/or colonial morphology (e.g. viridans streptococci or coagulase negative staphylococci, etc.). Refer to Supplementary Table S1 for complete classification details.

## Supplementary Information

Supplementary Table S1

Supplementary Figure S1
