# Supplementary material for: Comprehensive evaluation of complex polymicrobial specimens using next generation sequencing and standard microbiological culture
Source: Sci Rep. 2020 Mar 25;10:5446. doi: 10.1038/s41598-020-62424-x (PMC7096443; doi:10.1038/s41598-020-62424-x)
Supplement: Supplementary file 2 — Supplementary Figure S1 [file 41598_2020_62424_MOESM2_ESM.pdf]

Supplementary Figure S1. Heat maps showing the CRCs identified by NGS analysis for each specimen. Individual heat maps showing the CRCs identivied by NGS16S for each specimen are shown (19 total; no heatmap was generated for BAL18 because no organisms were identified for that specimen). Relative abundance of each classification group is shown by media type; relative abundance is indicated by color. DIR = NGS16S sequencing of DNA extracted directly from the sample; plate washes from media as described in Figure 1.

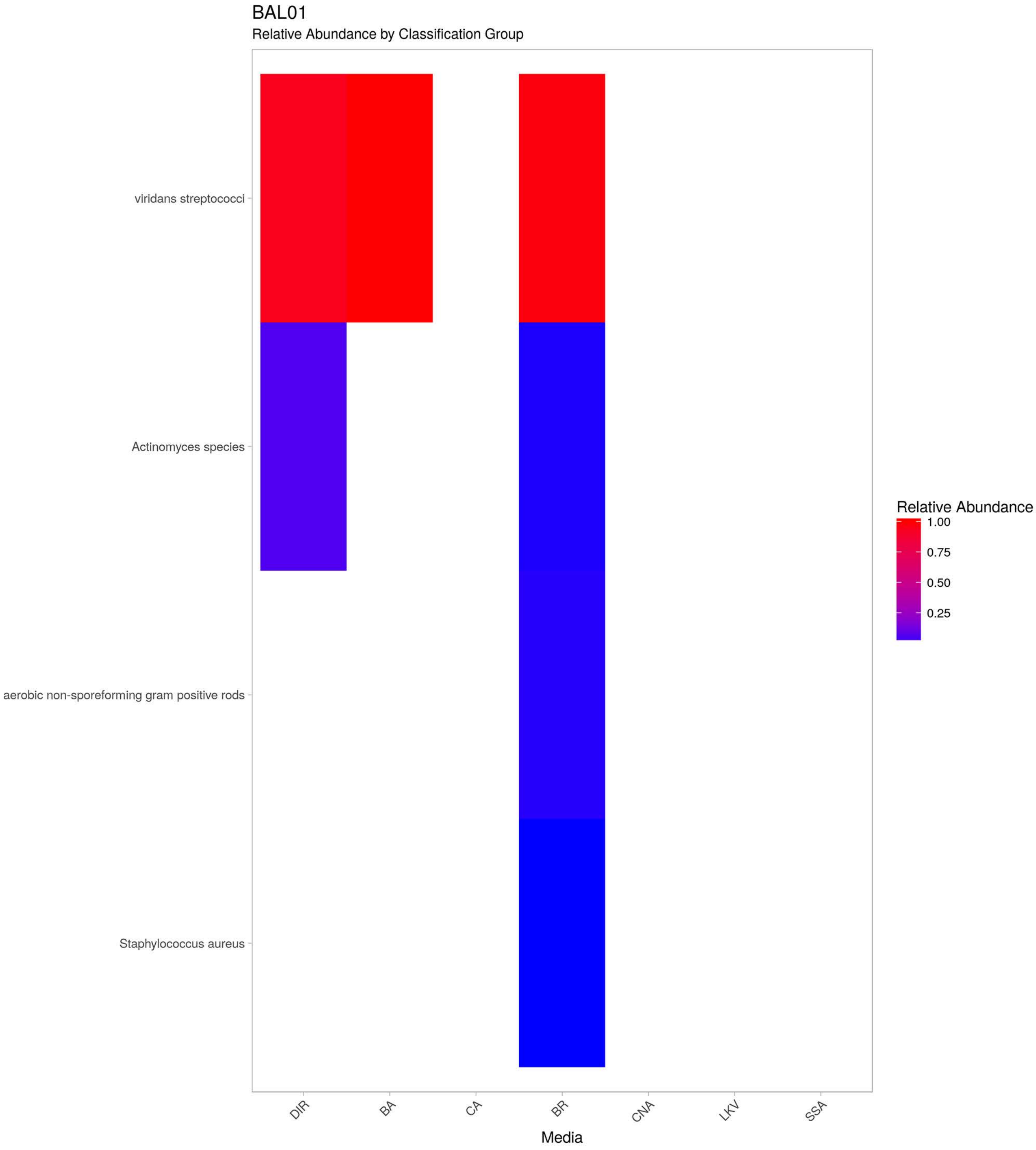

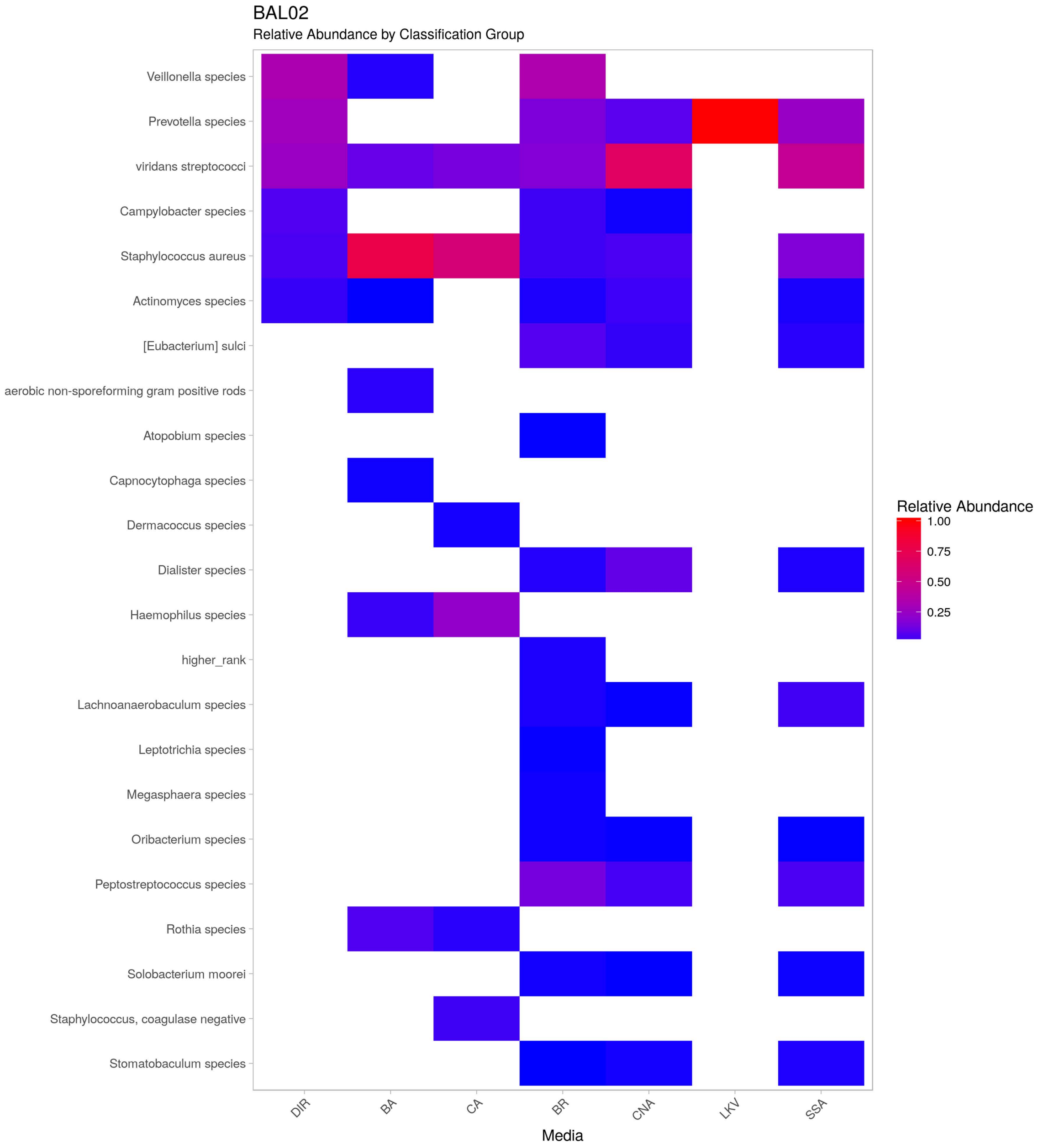

BAL02
Relative Abundance by Classification Group
Veillonella species
Prevotella species
viridans streptococci
Campylobacter species
Staphylococcus aureus
Actinomyces species
[Eubacterium] sulci
aerobic non-sporeforming gram positive rods
Atopobium species
Capnocytophaga species
Dermacoccus species
Dialister species
Haemophilus species
higher_rank
Lachnoanaerobaculum species
Leptotrichia species
Megasphaera species
Oribacterium species
Peptostreptococcus species
Rothia species
Solobacterium moorei
Staphylococcus, coagulase negative
Stomatobaculum species
DIR
BA
CA
BR
CNA
LKV
SSA
Media
Relative Abundance
1.00
0.75
0.50
0.25

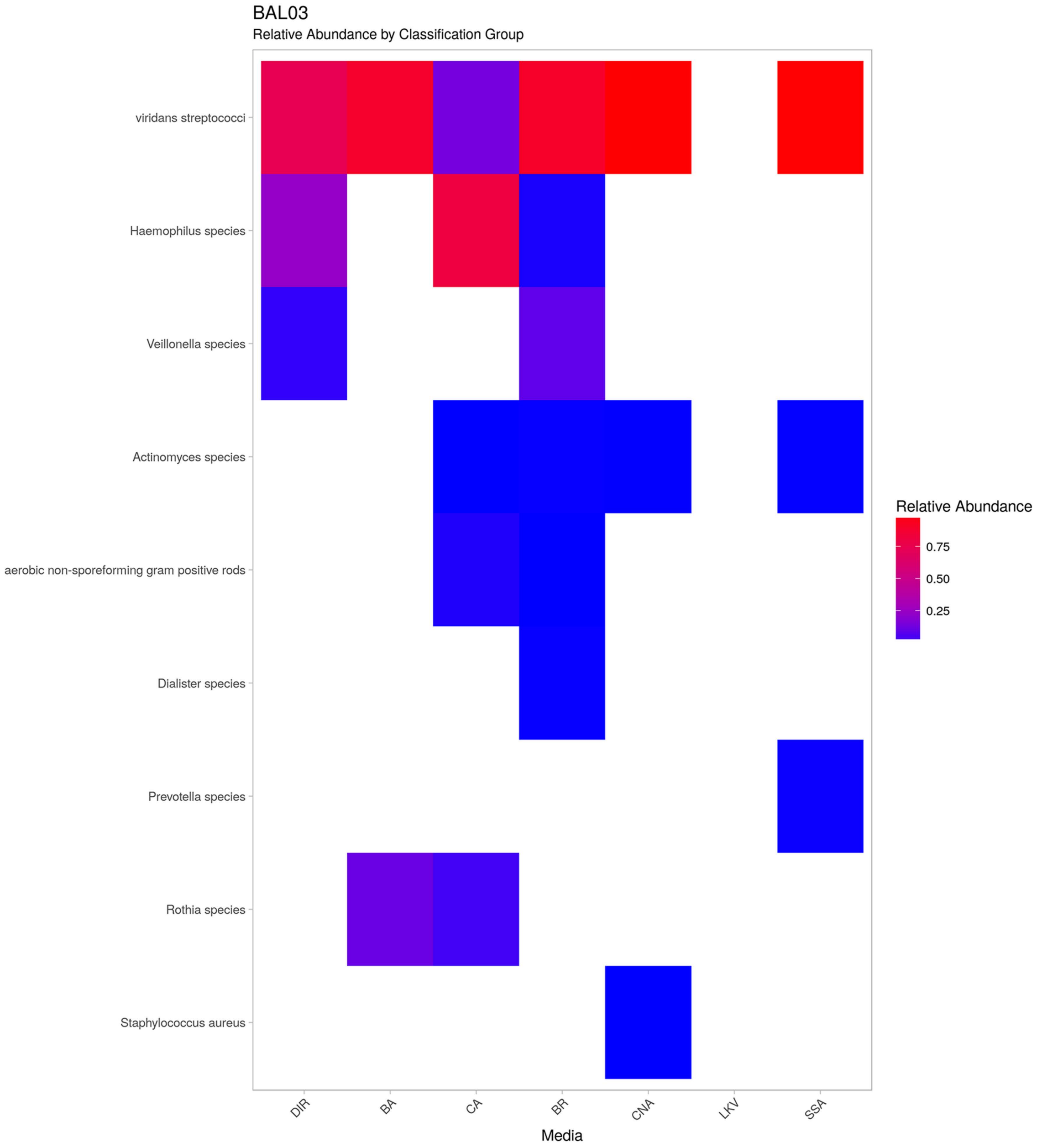
BAL03
Relative Abundance by Classification Group
viridans streptococci
Haemophilus species
Veillonella species
Actinomyces species
aerobic non-sporeforming gram positive rods
Dialister species
Prevotella species
Rothia species
Staphylococcus aureus
DIR
BA
CA
BR
CNA
LKV
SSA
Media
Relative Abundance
0.75
0.50
0.25

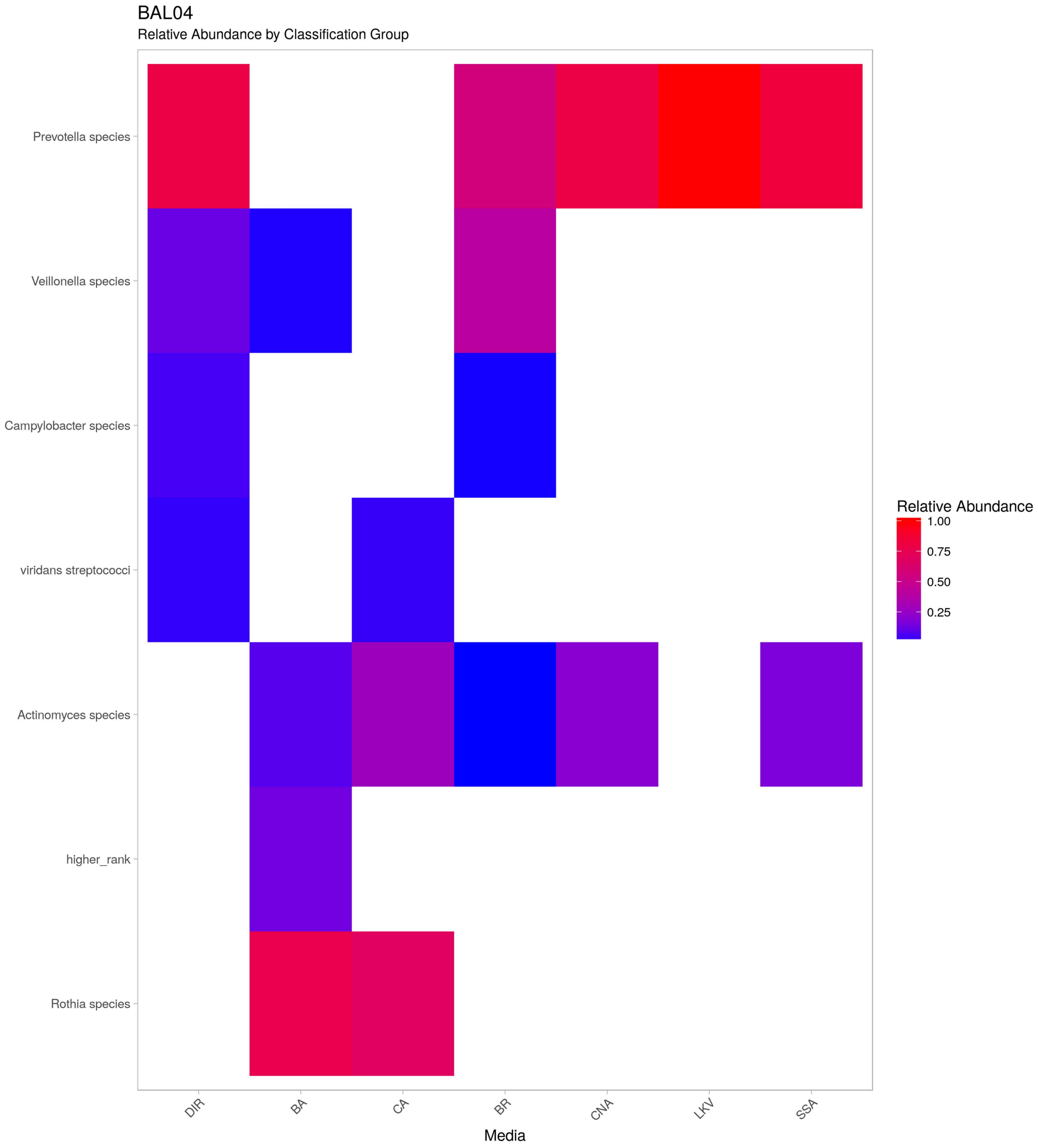

BAL04
Relative Abundance by Classification Group
Prevotella species
Veillonella species
Campylobacter species
viridans streptococci
Actinomyces species
higher_rank
Rothia species
DIR
BA
CA
BR
CNA
LKV
SSA
Media
Relative Abundance
1.00
0.75
0.50
0.25

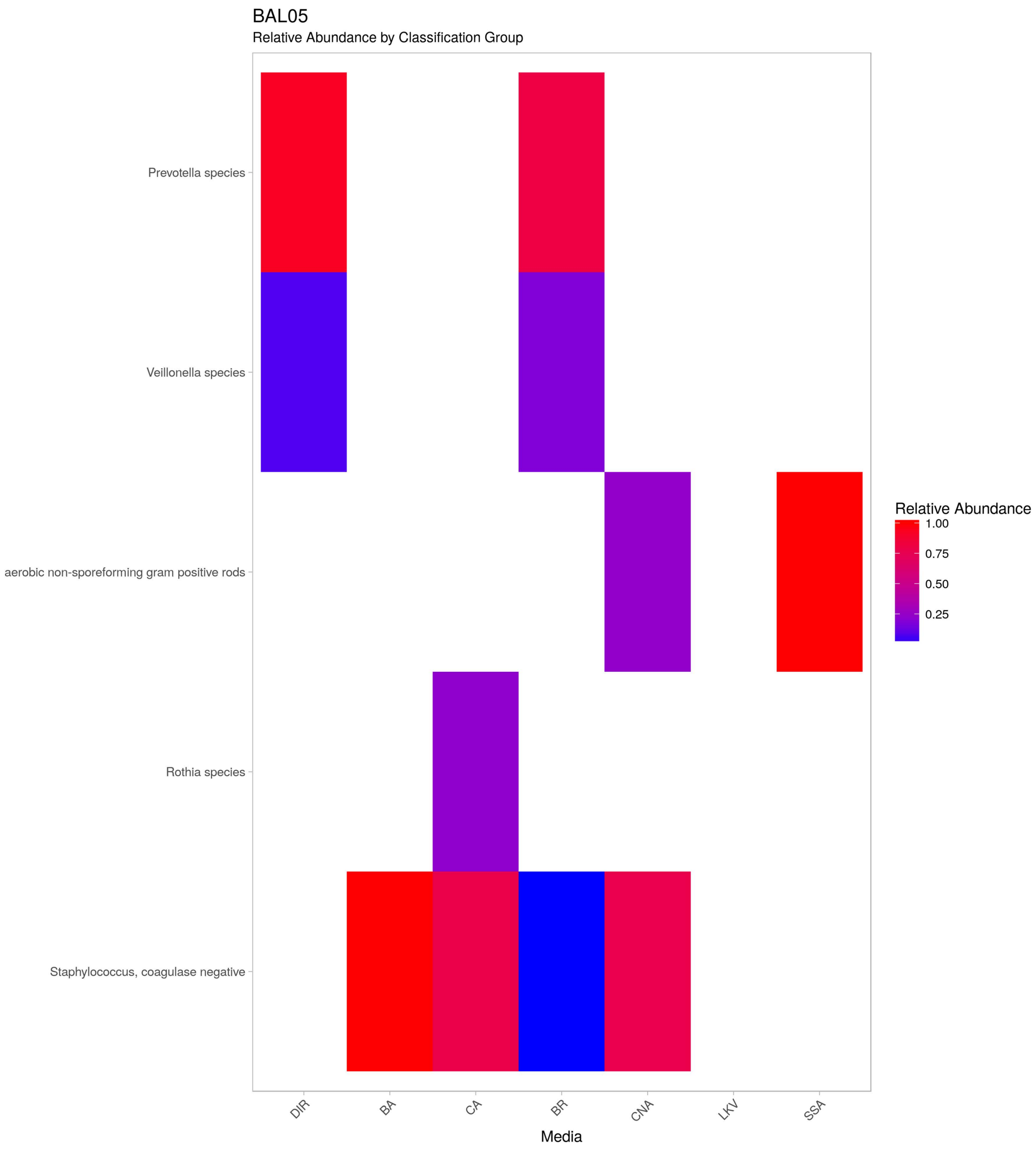

BAL05
Relative Abundance by Classification Group
Prevotella species
Veillonella species
aerobic non-sporeforming gram positive rods
Rothia species
Staphylococcus, coagulase negative
DIR
BA
CA
BR
CNA
LKV
SSA
Media
Relative Abundance
1.00
0.75
0.50
0.25

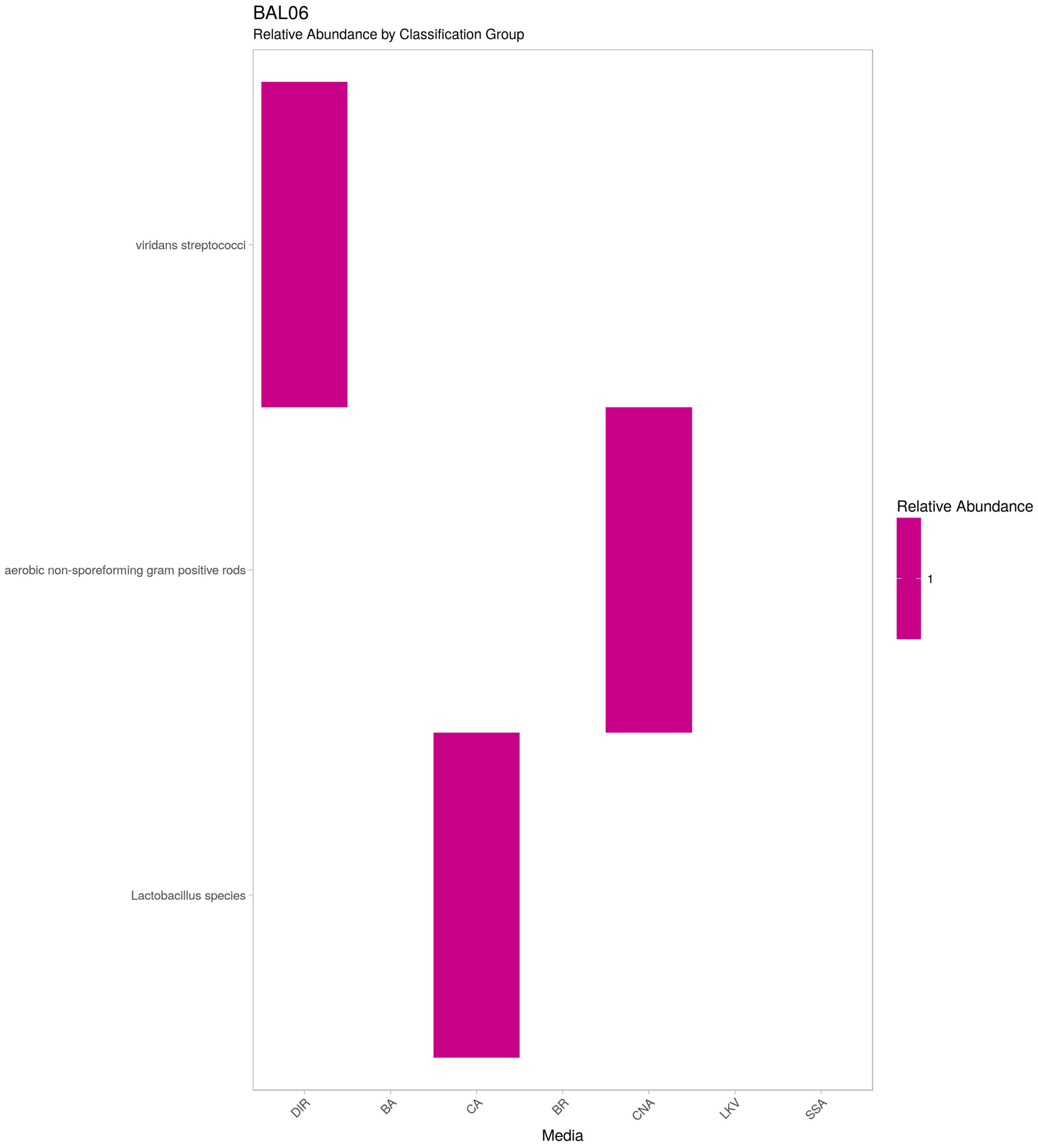

BAL06
Relative Abundance by Classification Group
viridans streptococci
aerobic non-sporeforming gram positive rods
Lactobacillus species
DIR
BA
CA
BR
CNA
LKV
SSA
Media
Relative Abundance
1

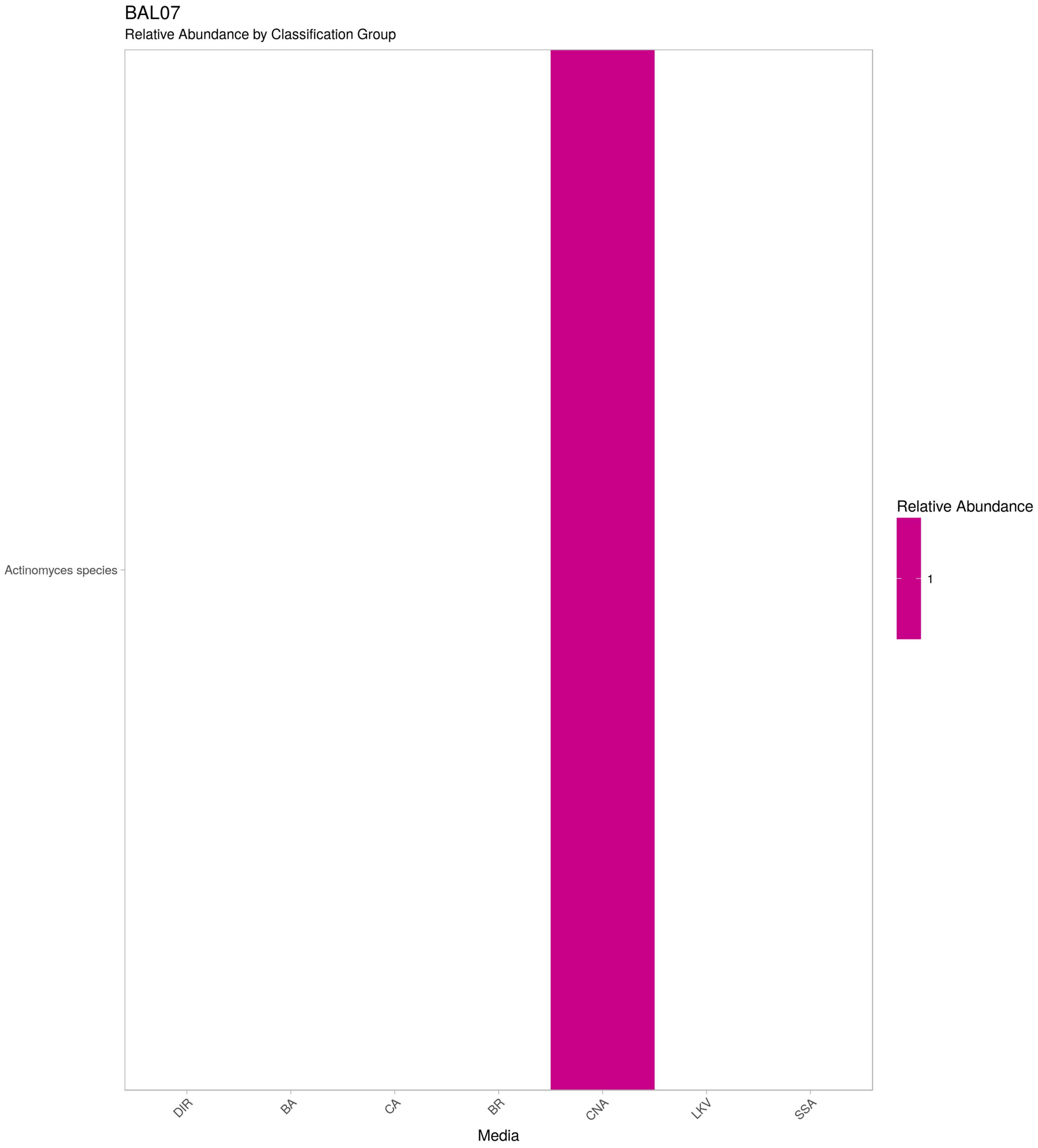
BAL07
Relative Abundance by Classification Group
Actinomyces species
DIR
BA
CA
BR
CNA
LKV
SSA
Media
Relative Abundance
1

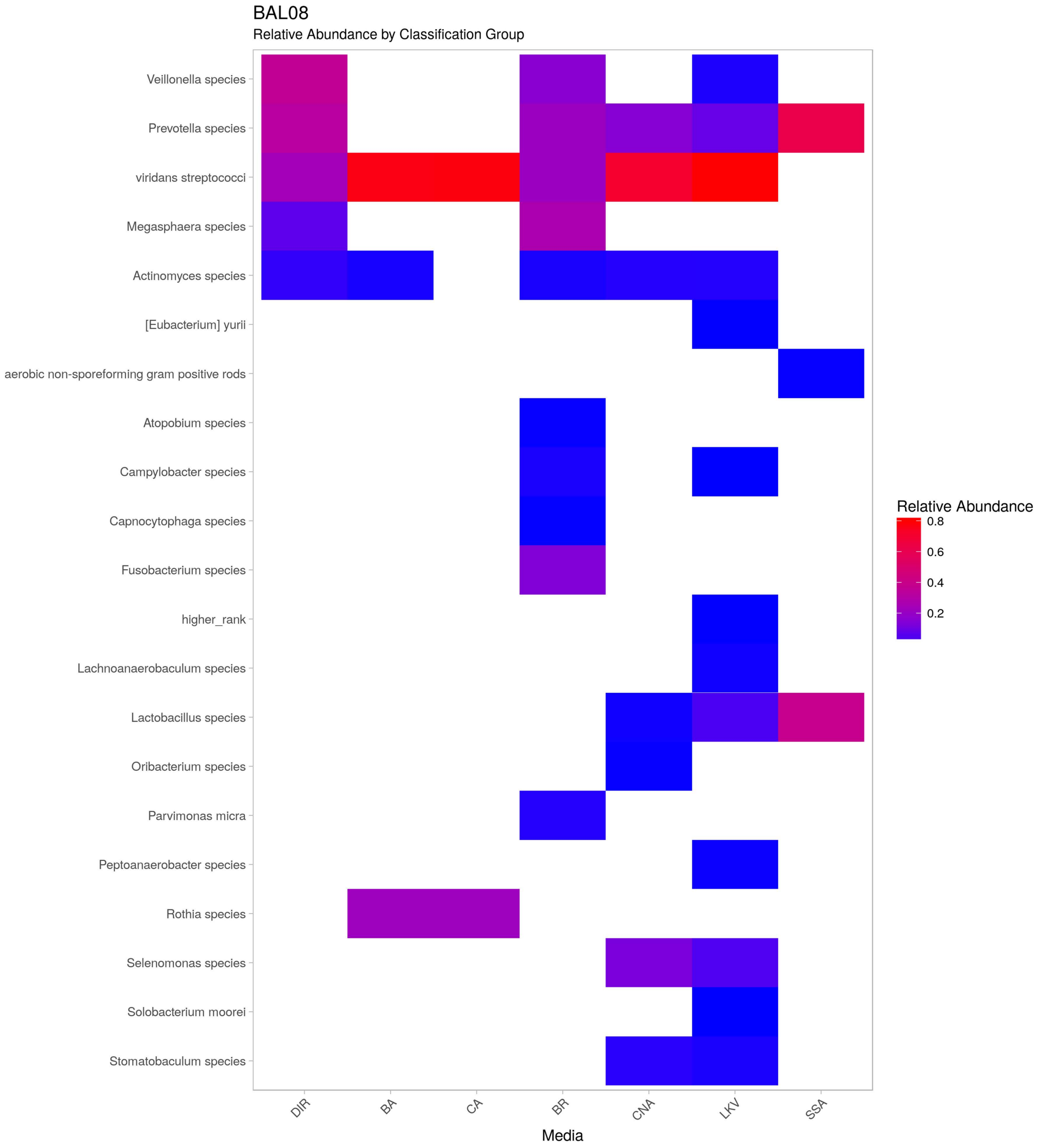
BAL08
Relative Abundance by Classification Group
Veillonella species
Prevotella species
viridans streptococci
Megasphaera species
Actinomyces species
[Eubacterium] yurii
aerobic non-sporeforming gram positive rods
Atopobium species
Campylobacter species
Capnocytophaga species
Fusobacterium species
higher_rank
Lachnoanaerobaculum species
Lactobacillus species
Oribacterium species
Parvimonas micra
Peptoanaerobacter species
Rothia species
Selenomonas species
Solobacterium moorei
Stomatobaculum species
DIR
BA
CA
BR
CNA
LKV
SSA
Media
Relative Abundance
0.8
0.6
0.4
0.2

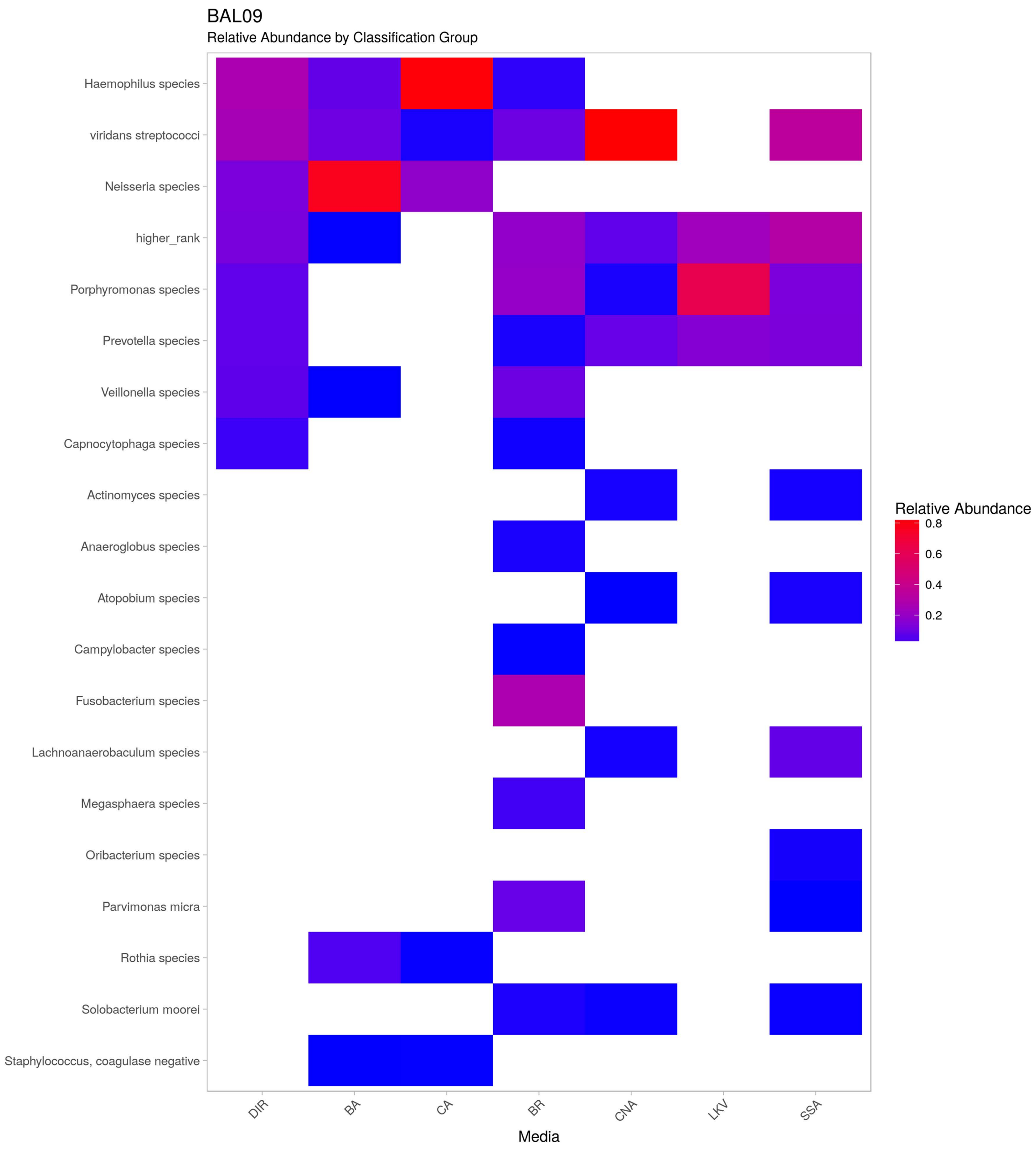
BAL09
Relative Abundance by Classification Group
Haemophilus species
viridans streptococci
Neisseria species
higher_rank
Porphyromonas species
Prevotella species
Veillonella species
Capnocytophaga species
Actinomyces species
Anaeroglobus species
Atopobium species
Campylobacter species
Fusobacterium species
Lachnoanaerobaculum species
Megasphaera species
Oribacterium species
Parvimonas micra
Rothia species
Solobacterium moorei
Staphylococcus, coagulase negative
DIR
BA
CA
BR
CNA
LKV
SSA
Media
Relative Abundance
0.8
0.6
0.4
0.2

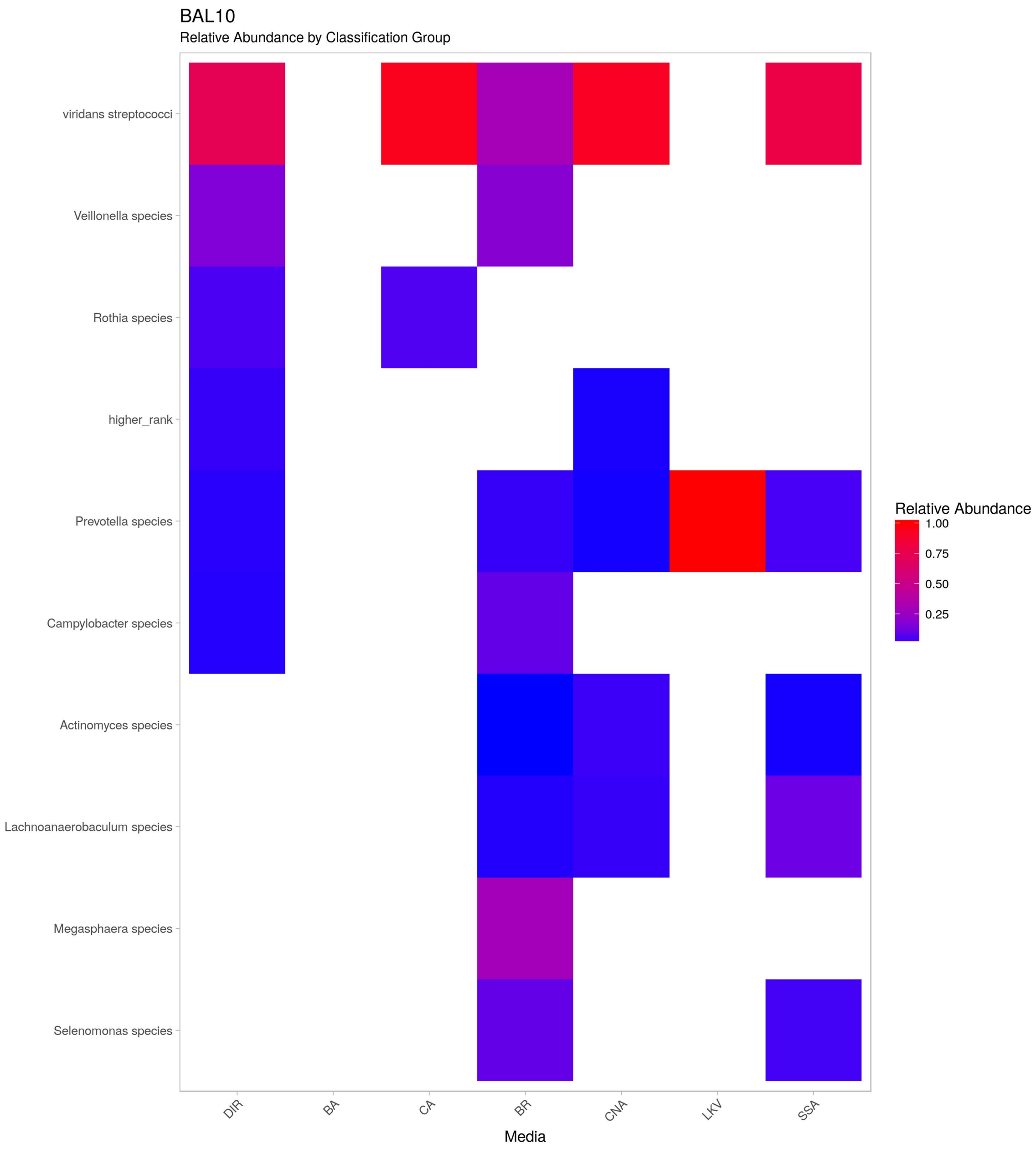
BAL10
Relative Abundance by Classification Group
viridans streptococci
Veillonella species
Rothia species
higher_rank
Prevotella species
Campylobacter species
Actinomyces species
Lachnoanaerobaculum species
Megasphaera species
Selenomonas species
DIR
BA
CA
BR
CNA
LKV
SSA
Media
Relative Abundance
1.00
0.75
0.50
0.25

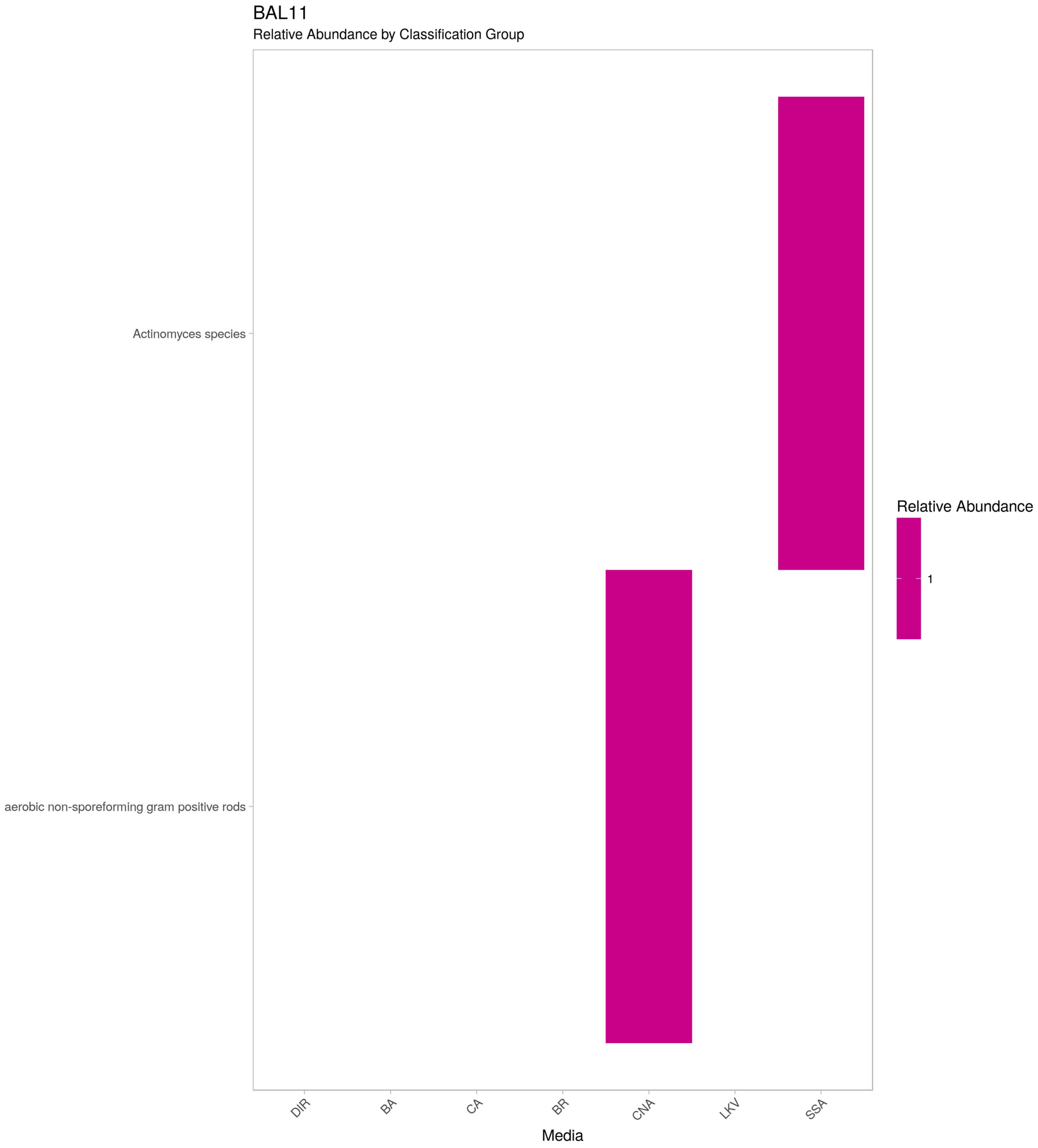
BAL11
Relative Abundance by Classification Group
Actinomyces species
aerobic non-sporeforming gram positive rods
DIR
BA
CA
BR
CNA
LKV
SSA
Media
Relative Abundance
1

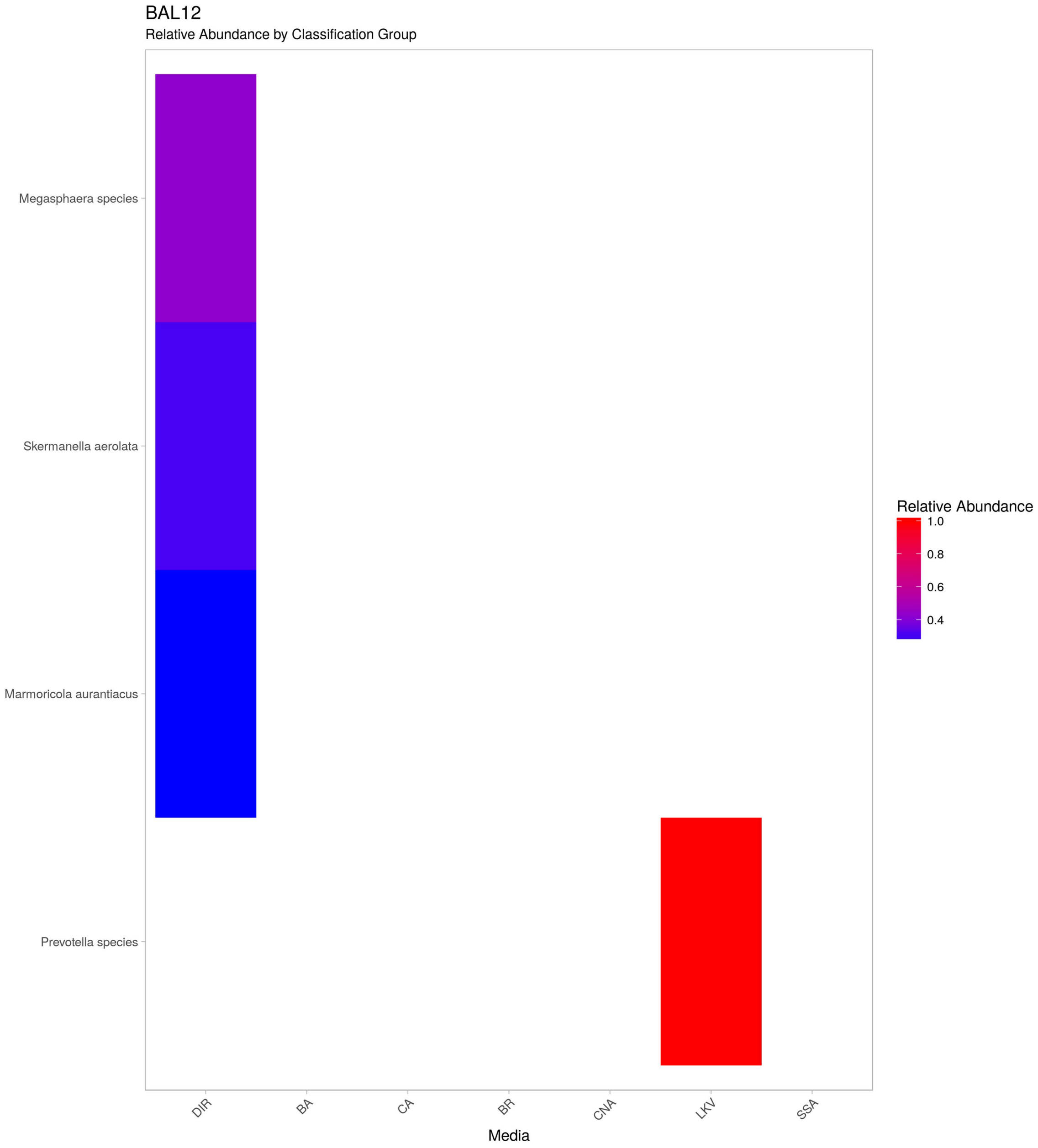
BAL12
Relative Abundance by Classification Group
Megasphaera species
Skermanella aerolata
Marmoricola aurantiacus
Prevotella species
DIR
BA
CA
BR
CNA
LKV
SSA
Media
Relative Abundance
1.0
0.8
0.6
0.4

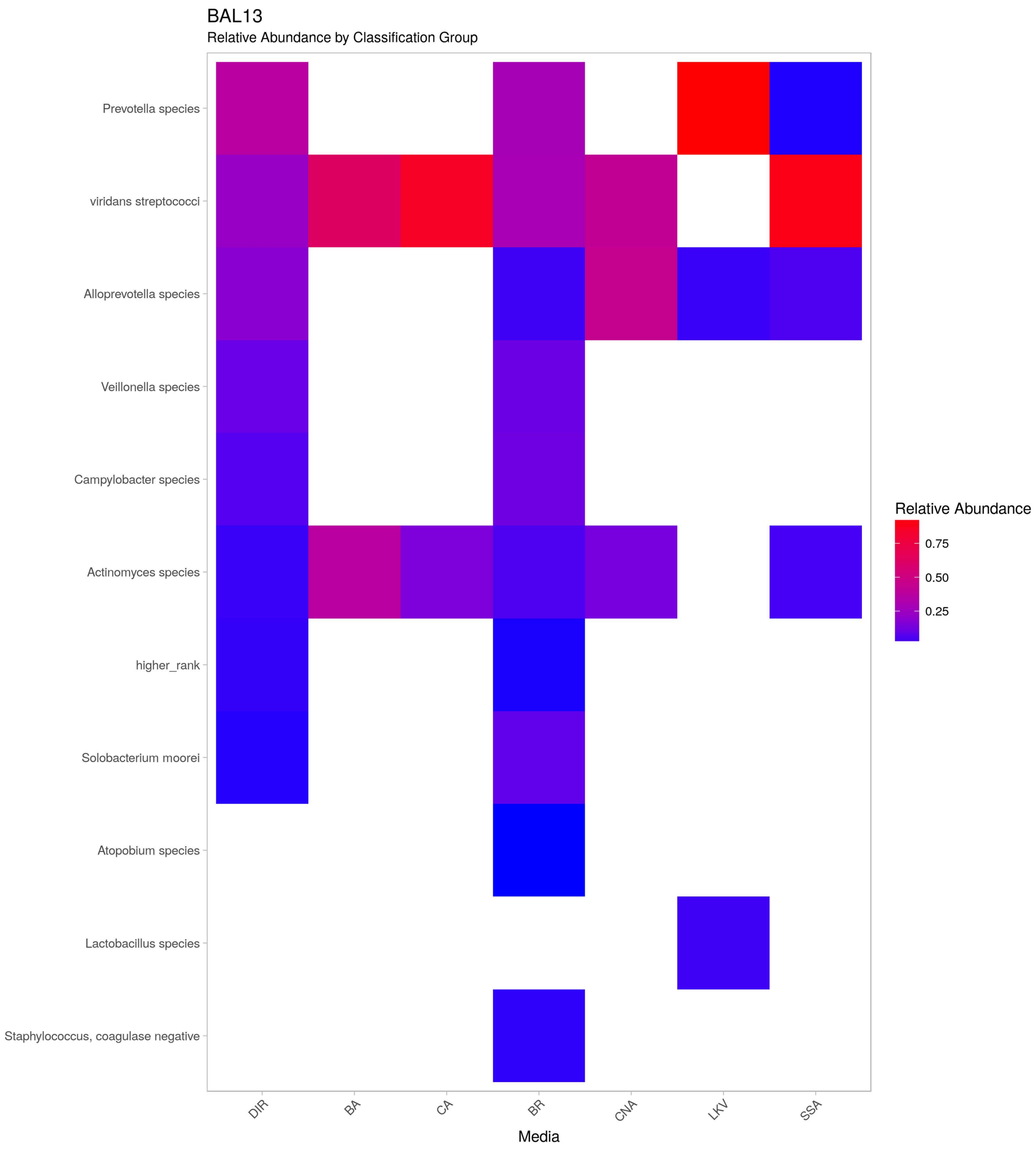

BAL13
Relative Abundance by Classification Group
Prevotella species
viridans streptococci
Alloprevotella species
Veillonella species
Campylobacter species
Actinomyces species
higher_rank
Solobacterium moorei
Atopobium species
Lactobacillus species
Staphylococcus, coagulase negative
DIR
BA
CA
BR
CNA
LKV
SSA
Media
Relative Abundance
0.75
0.50
0.25

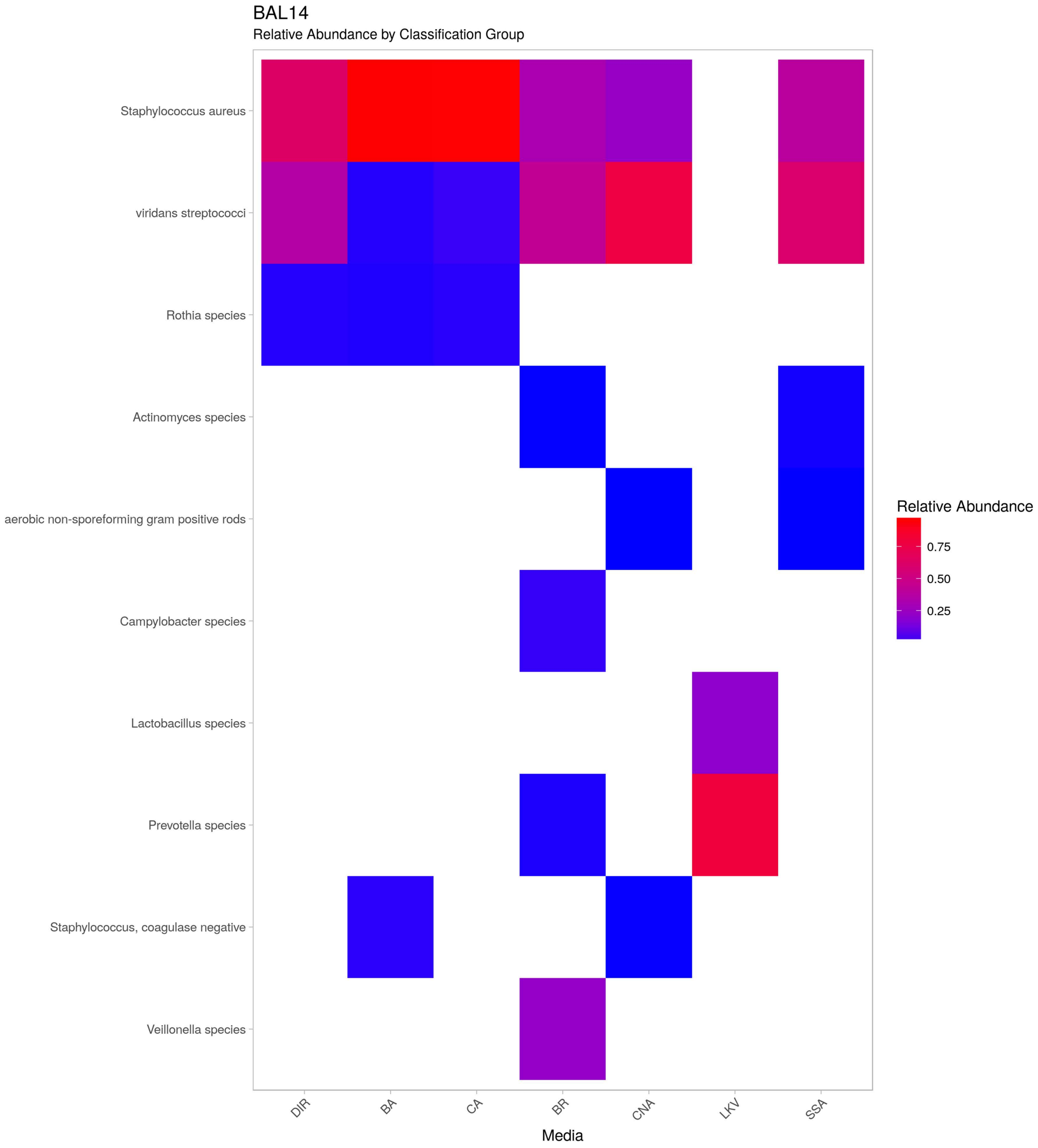
BAL14
Relative Abundance by Classification Group
Staphylococcus aureus
viridans streptococci
Rothia species
Actinomyces species
aerobic non-sporeforming gram positive rods
Campylobacter species
Lactobacillus species
Prevotella species
Staphylococcus, coagulase negative
Veillonella species
DIR
BA
CA
BR
CNA
LKV
SSA
Media
Relative Abundance
0.75
0.50
0.25

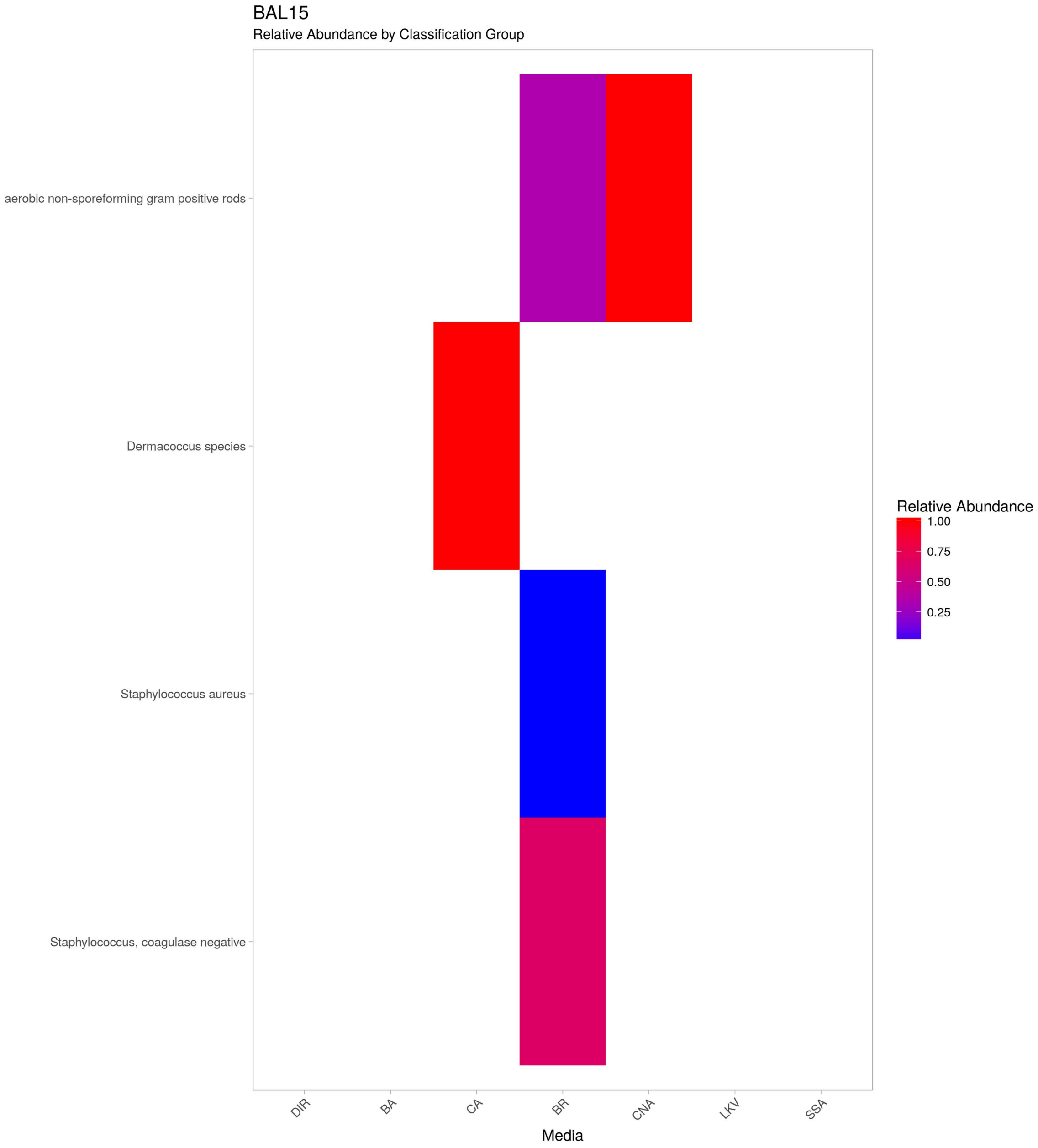

BAL15
Relative Abundance by Classification Group
aerobic non-sporeforming gram positive rods
Dermacoccus species
Staphylococcus aureus
Staphylococcus, coagulase negative
DIR
BA
CA
BR
CNA
LKV
SSA
Media
Relative Abundance
1.00
0.75
0.50
0.25

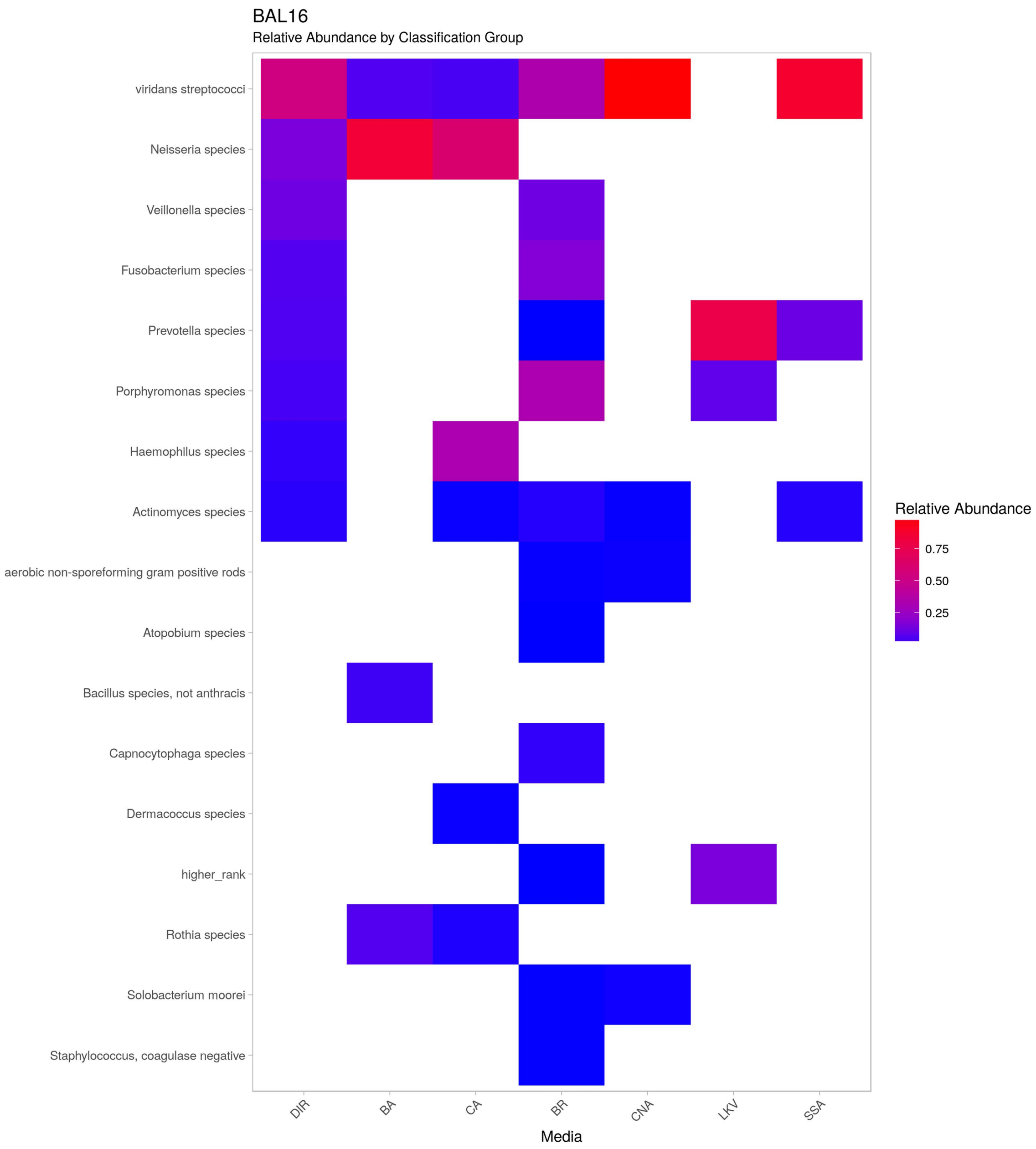
BAL16
Relative Abundance by Classification Group
viridans streptococci
Neisseria species
Veillonella species
Fusobacterium species
Prevotella species
Porphyromonas species
Haemophilus species
Actinomyces species
aerobic non-sporeforming gram positive rods
Atopobium species
Bacillus species, not anthracis
Capnocytophaga species
Dermacoccus species
higher_rank
Rothia species
Solobacterium moorei
Staphylococcus, coagulase negative
DIR
BA
CA
BR
CNA
LKV
SSA
Media
Relative Abundance
0.75
0.50
0.25

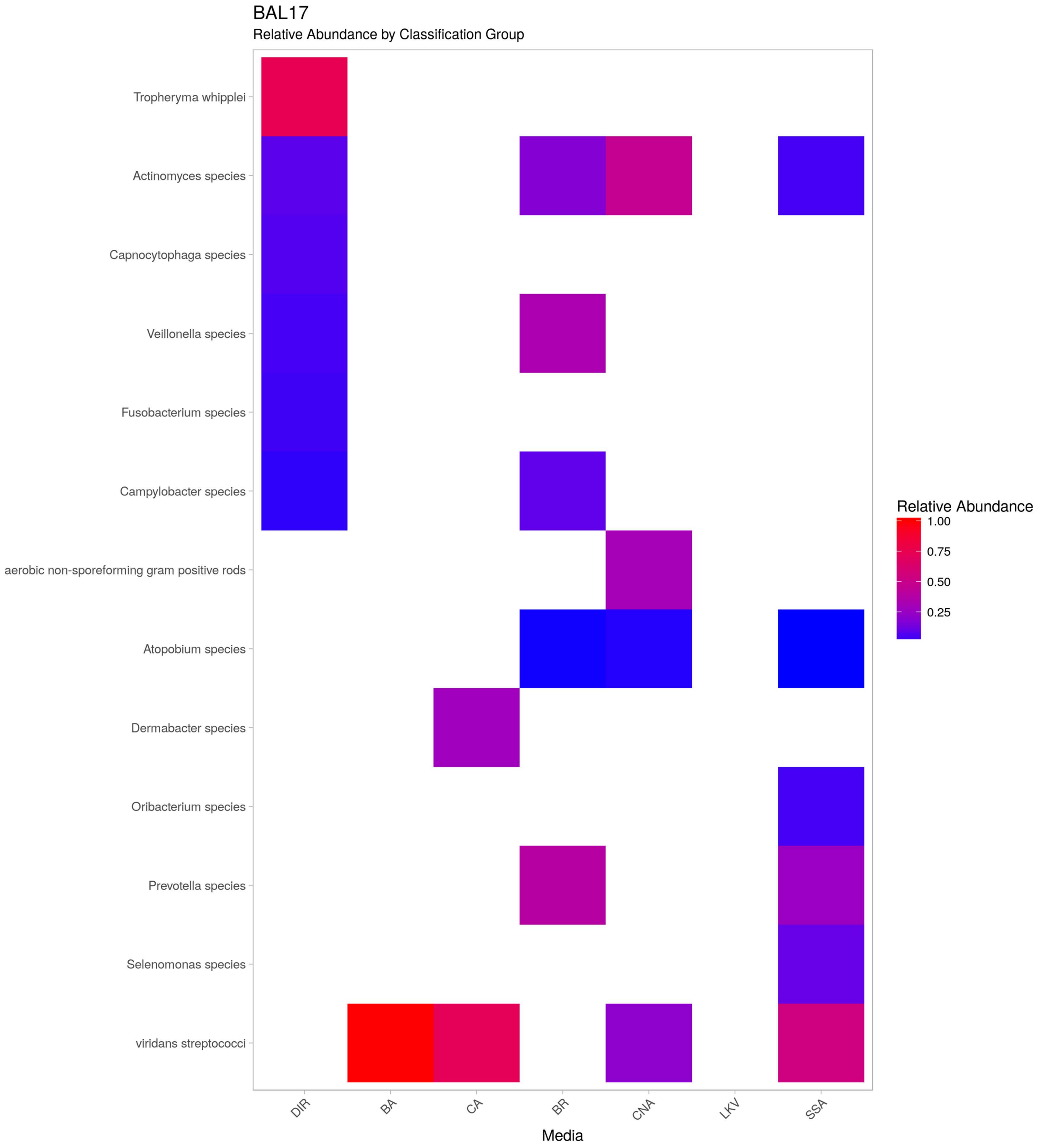
BAL17
Relative Abundance by Classification Group
Tropheryma whipplei
Actinomyces species
Capnocytophaga species
Veillonella species
Fusobacterium species
Campylobacter species
aerobic non-sporeforming gram positive rods
Atopobium species
Dermabacter species
Oribacterium species
Prevotella species
Selenomonas species
viridans streptococci
DIR
BA
CA
BR
CNA
LKV
SSA
Media
Relative Abundance
1.00
0.75
0.50
0.25

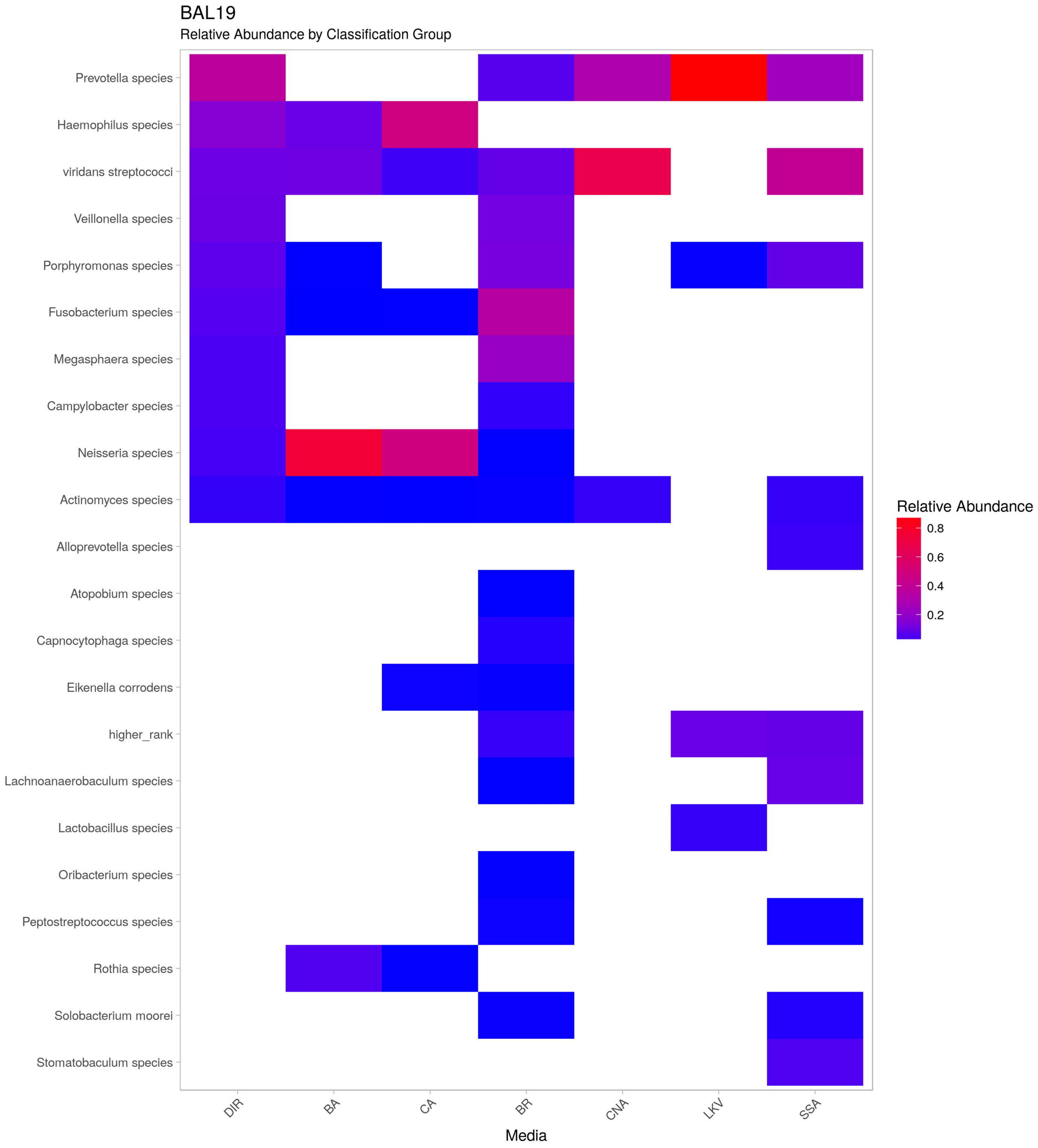
BAL19
Relative Abundance by Classification Group
Prevotella species
Haemophilus species
viridans streptococci
Veillonella species
Porphyromonas species
Fusobacterium species
Megasphaera species
Campylobacter species
Neisseria species
Actinomyces species
Alloprevotella species
Atopobium species
Capnocytophaga species
Eikenella corrodens
higher_rank
Lachnoanaerobaculum species
Lactobacillus species
Oribacterium species
Peptostreptococcus species
Rothia species
Solobacterium moorei
Stomatobaculum species
DIR
BA
CA
BR
CNA
LKV
SSA
Media
Relative Abundance
0.8
0.6
0.4
0.2

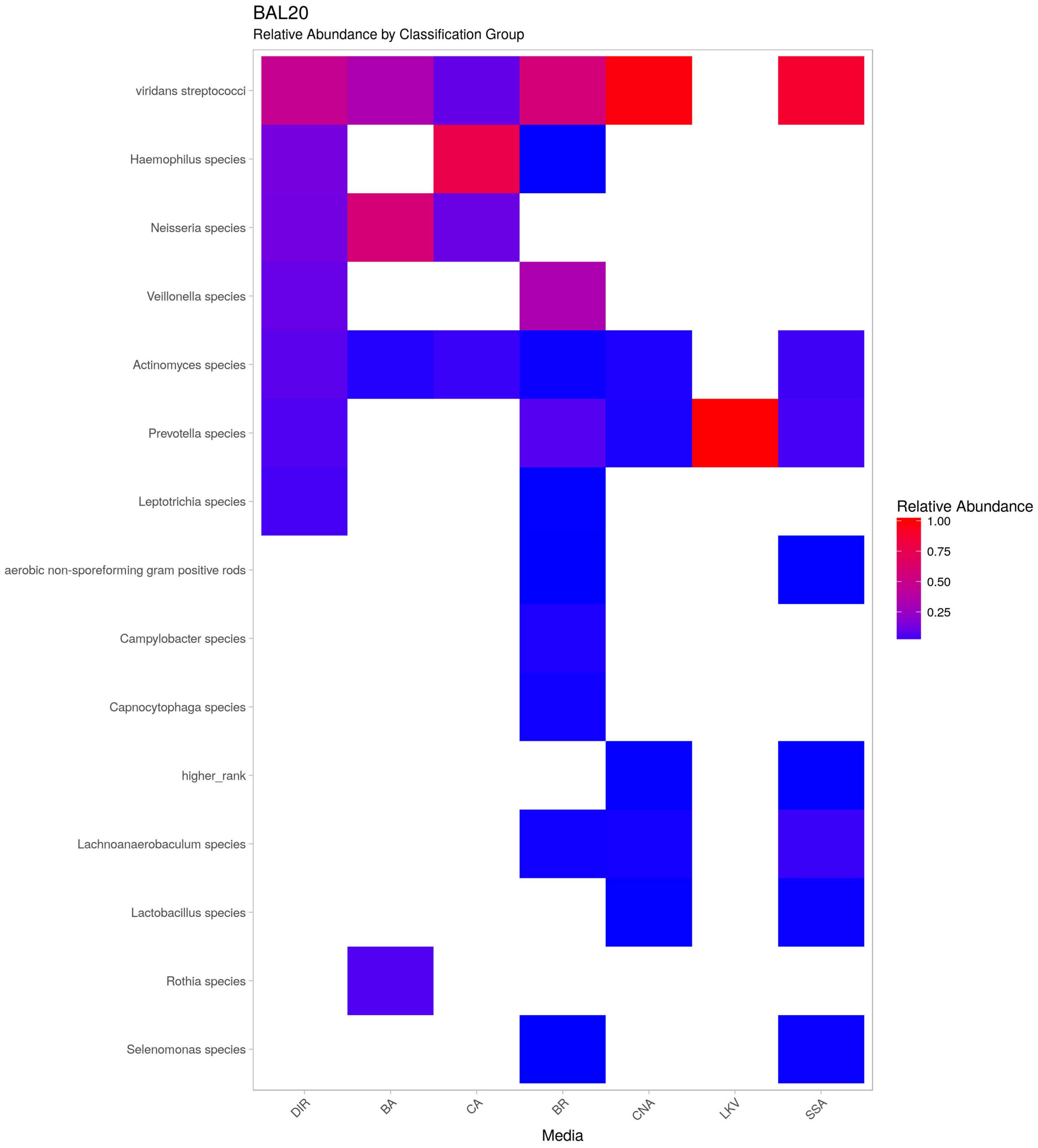
BAL20
Relative Abundance by Classification Group
viridans streptococci
Haemophilus species
Neisseria species
Veillonella species
Actinomyces species
Prevotella species
Leptotrichia species
aerobic non-sporeforming gram positive rods
Campylobacter species
Capnocytophaga species
higher_rank
Lachnoanaerobaculum species
Lactobacillus species
Rothia species
Selenomonas species
DIR
BA
CA
BR
CNA
LKV
SSA
Media
Relative Abundance
1.00
0.75
0.50
0.25
